# Combined Low-/High-Density Modern and Ancient Genome-Wide Data Document Genomic Admixture History of High-Altitude East Asians

**DOI:** 10.3389/fgene.2021.582357

**Published:** 2021-02-11

**Authors:** Yan Liu, Mengge Wang, Pengyu Chen, Zheng Wang, Jing Liu, Lilan Yao, Fei Wang, Renkuan Tang, Xing Zou, Guanglin He

**Affiliations:** ^1^ School of Basic Medical Sciences, North Sichuan Medical College, Nanchong, China; ^2^ Institute of Forensic Medicine, West China School of Basic Science and Forensic Medicine, Sichuan University, Chengdu, China; ^3^ Key Laboratory of Cell Engineering in Guizhou Province, Affiliated Hospital of Zunyi Medical University, Zunyi, China; ^4^ Center of Forensic Expertise, Affiliated Hospital of Zunyi Medical University, Zunyi, China; ^5^ Department of Forensic Medicine, College of Basic Medicine, Chongqing Medical University, Chongqing, China; ^6^ Department of Anthropology and Ethnology, Institute of Anthropology, National Institute for Data Science in Health and Medicine, and School of Life Sciences, Xiamen University, Xiamen, China

**Keywords:** 1240K dataset, ancient genomes, population history, forensic genetics, short tandem repeats, genetic polymorphism, East Asian highlander

## Abstract

The Tibetan Plateau (TP) is considered to be one of the last terrestrial environments conquered by the anatomically modern human. Understanding of the genetic background of highland Tibetans plays a pivotal role in archeology, anthropology, genetics, and forensic investigations. Here, we genotyped 22 forensic genetic markers in 1,089 Tibetans residing in Nagqu Prefecture and collected 1,233,013 single nucleotide polymorphisms (SNPs) in the highland East Asians (Sherpa and Tibetan) from the Simons Genome Diversity Project and ancient Tibetans from Nepal and Neolithic farmers from northeastern Qinghai-Tibetan Plateau from public databases. We subsequently merged our two datasets with other worldwide reference populations or eastern ancient Eurasians to gain new insights into the genetic diversity, population movements, and admixtures of high-altitude East Asians *via* comprehensive population genetic statistical tools [principal component analysis (PCA), multidimensional scaling plot (MDS), STRUCTURE/ADMIXTURE, *f_3_*, *f_4_*, *qpWave/qpAdm*, and *qpGraph*]. Besides, we also explored their forensic characteristics and extended the Chinese National Database based on STR data. We identified 231 alleles with the corresponding allele frequencies spanning from 0.0005 to 0.5624 in the forensic low-density dataset, in which the combined powers of discrimination and the probability of exclusion were 1–1.22E-24 and 0.999999998, respectively. Additionally, comprehensive population comparisons in our low-density data among 57 worldwide populations *via* the Nei’s genetic distance, PCA, MDS, NJ tree, and STRUCTURE analysis indicated that the highland Tibeto-Burman speakers kept the close genetic relationship with ethnically close populations. Findings from the 1240K high-density dataset not only confirmed the close genetic connection between modern Highlanders, Nepal ancients (Samdzong, Mebrak, and Chokhopani), and the upper Yellow River Qijia people, suggesting the northeastern edge of the TP served as a geographical corridor for ancient population migrations and interactions between highland and lowland regions, but also evidenced that late Neolithic farmers permanently colonized into the TP by adopting cold-tolerant barley agriculture that was mediated *via* the acculturation of idea *via* the millet farmer and not *via* the movement of barley agriculturalist as no obvious western Eurasian admixture signals were identified in our analyzed modern and ancient populations. Besides, results from the *qpAdm*-based admixture proportion estimation and *qpGraph*-based phylogenetic relationship reconstruction consistently demonstrated that all ancient and modern highland East Asians harbored and shared the deeply diverged Onge/Hoabinhian-related eastern Eurasian lineage, suggesting a common Paleolithic genetic legacy existed in high-altitude East Asians as the first layer of their gene pool.

## Introduction

East Asia, one of the oldest centers of plant and animal domestication, is home to almost one-quarter of the world’s population and encompasses substantial genetic, cultural, linguistic, and physical diversity. Understanding the peopling processes of East Asia or some unique harsh environment area is therefore of interest for elucidating how these extensive diversities arose and evolved. However, the comprehensive genetic history of East Asia is poorly understood due to the lack of ancient DNA from a denser genetic sampling or sparse sampling of modern East Asians and combined analyses of spatiotemporally diverse East Asian populations (Lu et al., 2016; Yao et al., 2017; Bai et al., 2018; He et al., 2020). Generally, patterns of genetic relatedness among present-day East Asians, especially for Han Chinese, run along a north-south cline (Qin et al., 2014; Chiang et al., 2018; Chen et al., 2019b; Gao et al., 2020b). Recent ancient genome-wide data of 26 ancient northern and southern East Asians (including Shandong Houli and Fujian Tanshishan cultural backgrounds) spanning 9,500–300 years ago indicated human population shifts and admixture in northern and southern China and confirmed the genetic division between northern and southern East Asians since early Neolithic (Yang et al., 2020). Wang et al. also reported genome-wide data from 383 modern and 191 ancient East Asians dating to around 6,000 BCE–1,000 CE and illuminated the dispersal models of the ancestors of Mongolic, Tungusic, Sino-Tibetan, Austronesian, Tai-Kadai, and Austroasiatic languages and showed the complex population interactions among different ancient East Asians (Wang et al., 2020). Additionally, Ning et al. reported 55 ancient genomes dating to 7,500–1,700 years ago from the Yellow River (Henan Yangshao, Longshan, and Shangzhou cultures and Qinghai Qijia culture), West Liao River (Hongshan and Xiajiadian cultures), and Amur River (Haminmangha culture) basins and illustrated a link between changes in subsistence strategy and human activities (migration and admixture; Ning et al., 2020). However, these ancient genomes from the lowland East Asians showed a finer-scale landscape of population origin, diversification, and admixture in the lowland regions, and the population genetic admixture history of the highland region kept underrepresented and unclear due to the sparse genetic sampling of modern and ancient populations from the Qinghai-Tibet Plateau, which impedes our ability to connect temporally and geographically dispersed ancient East Asians and modern Tibetans.

The Qinghai-Tibet Plateau, also called the Tibetan Plateau (TP), a high-altitude arid steppe bounded by the world’s tallest mountains, represents one of the most challenging environments with low temperature and hypobaric hypoxia for human settlement. As one of the last populated areas occupied by modern humans, the exact timing of the peopling of the TP and the migration trajectories of Tibetans have appealed to growing academic interests. The recovered paleoproteomic results of a Xiahe Denisovan mandible from the TP indicated that archaic hominins occupied the TP in the Middle Pleistocene epoch and successfully adapted to the high-altitude environments with the accumulation of *Endothelial PAS domain protein 1* (*EPAS1*) adaptive alleles (Chen et al., 2019a). Archeological investigations documented that the earliest modern human foraging of the TP may have begun at least ~40 to 30 thousand years ago (kya; Zhang et al., 2018). Considerable progress on the anthropological, archeological, and genetic perspectives of archaic and modern humans provided the conclusive evidence in support of the Paleolithic initial peopling of the TP and indicated that the permanent human occupation had taken place around 3.6 kya, which was most likely facilitated by the spread of barley/wheat-based agriculture (Qi et al., 2013; Chen et al., 2015; Lu et al., 2016; Meyer et al., 2017; Li et al., 2019b; Gao et al., 2020a; Ren et al., 2020). The matrilineal evidence revealed Tibetan-prevailing lineages of A11a1a and M9a1a1c1b1a and demonstrated that the ancestry of Tibetans could largely be traced back to the Neolithic millet farmers from northern China (Zhao et al., 2009; Qin et al., 2010; Qi et al., 2013; Li et al., 2019b; Wang et al., 2020). The coalescence ages of Tibetan-specific Y-chromosomal lineages served as another strong evidence that the earlier settlers on the TP could have survived in the Last Glacial Maximum (LGM) and contributed to the gene pool of present-day Tibetan populations (Qi et al., 2013). It also revealed that Neolithic expansions of low-altitude agriculturalists had a prominent impact on the genomic makeup of modern Tibetans (Qi et al., 2013). Besides, genome-wide data revealed a relatively closer genetic affinity between Tibetans and Han Chinese and indicated that Tibetans arose from a mixture of multiple ancestral gene pools and most of the Tibetan gene pool could be attributed to the post-LGM arrivals of Neolithic ancestry (Qi et al., 2013; Lu et al., 2016; Yao et al., 2017). Linguistic study from the Bayesian phylogenetic analysis of the Sino-Tibetan language family has suggested that the Tibeto-Burman-speaking populations diverged from Han Chinese with an average coalescence age of approximately 5.9 kya (Zhang et al., 2019). Furthermore, genetic observations based on forensically related markers also revealed the consistent phylogenetic relationships between Tibetan and other geographically or ethnically different groups (He et al., 2018a,b; Wang et al., 2018b, 2020; Zou et al., 2018; Li et al., 2019a).

Taken together, current archeological, anthropological, genetic, and linguistic findings suggested that the initial Paleolithic occupation of the TP combined with later multiple migrations at different times and from different regions may have created the complicated and mosaic demographic history of Tibetans. However, available genetic data are insufficient to address the discrepancy between demographic history constructed by different regional studies and hamper the exploration of genetic variations of Tibetans based on the forensically related markers. Hence, extending the existing forensic reference database and dissecting the genetic differentiation among different Tibetan groups or between Tibetans and other reference populations based on the combined resolution of modern and ancient genomes is indispensable. Here, we mainly aimed to focus on the following topics: (I) explore the pattern of genetic diversity of highland East Asian based on short tandem repeat (STR) and single nucleotide polymorphism (SNP) data; (II) dissect the potential gene flow events between highland Tibetan-Burman speakers and close lowland East Asian populations; (III) explore whether there is a genetic continuity between modern Highlanders and ancient populations who were linked *via* the archeologically attested similarities of cultures from Nepal and upper Yellow River (Qijia people) and further explore the extent to which it was mediated *via* the population movement though a northeast geographical corridor; and (IV) evaluate to what extent of the barley/wheat agriculture spread in the Ganqing region was mediated *via* cultural diffusion or demic diffusion from the Fertile Crescent.

## Materials and Methods

### Sample Collection

**Figure 1:**
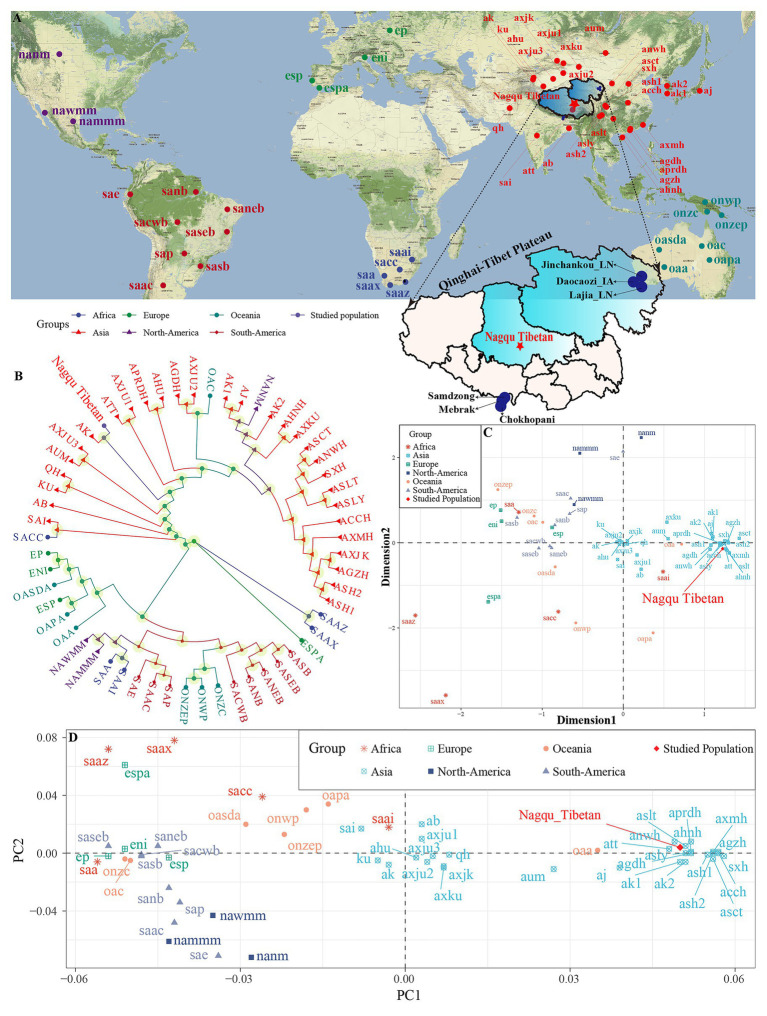
Sampling geographical region and patterns of the genetic relationship between Tibetan and worldwide reference populations based on the STR low-density dataset. **(A)** Geographical position of Nagqu City and Qinghai-Tibet Plateau and other included reference modern and ancient populations. **(B)** Phylogenetic relationship between Nagqu Tibetan and other 56 worldwide reference populations based on the pairwise genetic distance. **(C)** Genetic relationship between Nagqu Tibetan and other 56 worldwide reference populations revealed by the multidimensional scaling plots. **(D)** Two-dimensional scaling plots of the top two components in PCA analysis. The full population names (codes) are submitted in Supplementary Table S1.

Here, we carried out the present study in 1,089 unrelated Tibetan individuals (593 males and 496 females) residing in Nagqu – the northeastern prefecture-level city of Tibet Autonomous Region (Figure 1A). All participants enrolled in the present study have signed the written informed consent form and are required to be the indigenous Tibetan people. Bloodstains were collected from people with no mixed marriage with people of other ethnic groups. This project was performed in accordance with the recommendations of the Declaration of Helsinki (Nicogossian et al., 2014) and approved by the Ethics Committees of North Sichuan Medical College and Zunyi Medical University.

### DNA Extraction, Quantification, and Genotyping

Human genomic DNA was extracted using the PureLink Genomic DNA Mini Kit (Thermo Fisher Scientific) and quantified by employing the NanoDrop-2000 on the basis of the manufacturer’s instructions. Twenty autosomal short tandem repeats (A-STRs) recommended by the Chinese National Database (CND) as well as two gender-determining genes (Amelogenin and Y-indel) were amplified simultaneously using the STRtyper-21G PCR assay on a ProFlex PCR System (Thermo Fisher Scientific) following the manufacturer’s recommendation. ABI 3130 Genetic Analyzer (Thermo Fisher Scientific) was utilized to separate the PCR products, and the GeneMapper ID-X v.1.4 software was used to visualize the electrophoresis results.

### Data Analysis

#### Analysis of Genetic Variations Based on Low-Density STRs

The online software of the STR Analysis for Forensics (STRAF; Gouy and Zieger, 2017) was adopted to evaluate the allelic frequencies and forensic statistical parameters of 20 A-STRs. The exact tests of linkage disequilibrium (LD) and Hardy-Weinberg equilibrium (HWE), as well as evaluation of the heterozygosity indexes (observed heterozygosity: Ho; and expected heterozygosity: He), were conducted using the Arlequin v.3.5.2.2 (Excoffier and Lischer, 2010). Nei’s pairwise genetic distances between Nagqu Tibetan and 56 worldwide reference populations were estimated *via* the Gendist package implemented in the PHYLIP v.3.695 (Retief, 2000) and imported into R software^1^ for heatmap plotting. Frequency-based principal component analysis (PCA) of the 17 A-STRs among 57 worldwide populations (the detailed codes of population information is listed in Supplementary Table S1 and Figure 1A) was carried out using the Multivariate Statistical Package (MVSP) software v.3.22 (Kovach, 2013). The Nei’s distance matrix was then applied to perform the multidimensional scaling (MDS) analysis using the IBM SPSS v.21.0 and reconstruct a neighbor-joining (NJ) tree *via* the Molecular Evolutionary Genetics Analysis v.7.0 (Mega 7.0; Kumar et al., 2016). Furthermore, we employed the STRUCTURE v.2.3.4.21 (Evanno et al., 2005) to dissect the genetic similarity among 3,287 individuals from 11 Chinese populations with *K* values ranging from 2 to ~6 under the “correlated allele frequencies” and “LOCPRIOR” models.

#### High-Density Genome-Wide Data Analysis

We retrieved 1,233,013 SNPs of Tibetan and Sherpa from the Simons Genome Diversity Project (Mallick et al., 2016); ancient Tibetan genome-wide SNP data from eight Nepal individuals (Jeong et al., 2016) with cultural backgrounds of Chokhopani, Samdzong, and Mebrak; and 11 late Neolithic to Iron Age from the northeastern edge of the TP (Ning et al., 2020). We then merged the aforesaid data with other publicly available data of modern and ancient East Asians (Patterson et al., 2012; Yang et al., 2017, 2020; Lipson et al., 2018; Jeong et al., 2019; Liu et al., 2020; Ning et al., 2020). The geographical position and corresponding archeological periods are provided in Supplementary Figure S1. We then pruned SNPs in strong linkage disequilibrium by applying PLINK v.1.9 (Chang et al., 2015) with parameters of --indep-pairwise 200 25 0.4. We performed model-based clustering analysis using the ADMIXTURE v.1.3.0 (Alexander et al., 2009) with the 10-fold cross-validation (--cv = 10), presupposing the number of *K* values ranging from 2 to 8 in 100 bootstraps with different random seeds. The caution is that the clusters obtained in this model-based analysis are only “similarity” measures based on complex algorithms, and individuals are assigned to a cluster in whole or in part, which can be used to explore the genetic similarities and differences based on the shared components among then. We computed Fst values using the EIGENSOFT with the default parameters of inbreed: YES and fstonly: YES.

We computed outgroup *f_3_-statistics* using the *qp3Pop* program of the ADMIXTOOLS package (Patterson et al., 2012) and looked for evidence of maximized shared genetic drift. We also conducted admixture *f_3_-* and *f_4_-statistics* using the *qp3Pop* and *qpDstat* packages from the same program with the default parameters to assess the potential admixture signals from different source populations into the targeted populations. We calculated standard errors using the weighted block jackknife approach.

Applying the covariance of the allele frequency profiles as input, we ran TreeMix v.1.13 (Pickrell and Pritchard, 2012) with migration events varying from 0 to 8 to generate the topology with the maximum likelihood. Based on the results of the *f-statistics*, admixture graph modeling was carried out using the *qpGraph* software as implemented in the ADMIXTOOLS using central African of Mbuti as an outgroup (Fu et al., 2015). We applied the programs *qpWave* and *qpAdm* from the ADMIXTOOLS to model the targets as a combination of putatively selected source populations and estimate the ancestry proportions by solving a matrix of *f_4_-statistics* (Haak et al., 2015). We used a batch of outgroups and basic phylogenetic relationships followed Wang’s model (Wang et al., 2020), which represented modern and ancient global genomic variations and provided a good resolution for distinguishing Tibetan Highlanders.

## Results

### Genetic Diversity, Forensic Features, and STR-Based Population Comparisons

We genotyped 20 autosomal STRs and two Y-linked genetic loci for sex determination in 1,089 unrelated Nagqu highland Tibetans using the new generation of STRtyper-21G PCR amplification system. As displayed in Supplementary Table S2, one out of 20 STR loci (D3S1358) was de*via*ted from the HWE after applying the Bonferroni correction (0.05/20 = 0.0025), and LD was observed in the locus pair of TPOX-Penta E (Supplementary Table S3, 0.00020) after conducting the multiple tests of Bonferroni correction (0.05/190 = 0.00026). A total of 231 alleles were identified with the corresponding allelic frequencies spanning from 0.0005 to 0.5624 (Supplementary Table S4). The values of Ho and He, as well as forensic parameters, including discrimination power (DP), probability of exclusion (PE), and typical paternity index (TPI) are presented in Supplementary Table S2. The Ho varied from 0.6217 to 0.9183, and the He spanned from 0.6038 to 0.9182. The measured values of DP and PE were in the range of 0.7854–0.9865 and 0.3177–0.8329, respectively. The value TPI varied from 1.3216 to 6.1180. Additionally, the combined power of discrimination (CPD) value reached 1–1.22E-24 in Nagqu Tibetan, and the value of the combined probability of exclusion (CPE) was 0.999999998.

We explored the genetic relationships between Nagqu Tibetan and other 56 reference populations *via* the pairwise genetic distances, PCA, MDS, and NJ tree. The pairwise genetic distances among 57 populations are listed in Supplementary Table S5 and Supplementary Figure S2. The Chengdu Tibetan (ASCT) was identified as the genetically closest population to Nagqu Tibetan (0.012), followed by Liangshan Tibetan (ASLT, 0.0134) and Liangshan Yi (ASLY, 0.0146). The African AmaXhosa (SAAX) shows the largest genetic differences with Nagqu Tibetan (0.2097). Subsequently, MDS and NJ tree (Figures 1B,C) were depicted based on the pairwise genetic distance matrix. On the NJ tree (Figure 1B), all 57 worldwide populations were roughly grouped into two clades: Asian groups and other continental groups. It is interesting to find that the Nagqu Tibetan first clustered with Akto Kyrgyz (AK) and then clustered with Tibet Tibetan (ATT). There needed to be more caution that the NJ-based bifurcating tree just provided the basic framework of population relationship not only due to an NJ tree is an approximation to a fully additive tree but also the fitting process ignored the potential exited admixture events. Thus, TreeMix and *qpGraph*-based phylogenetic relationship reconstruction needed to be conducted and will be discussed in detail in the following contents. As displayed in Figure 1C, the Asian populations clustered close to each other, which can be further grouped into Sino-Tibetan (ST) cluster and Altai-Turkic (AT) cluster, and the North/South American populations formed a relatively looser cluster. Conversely, other continental populations were scattered in the left and lower right quadrants. Nagqu Tibetan was located close to Tibet Tibetan (ATT) and Liangshan Tibetan (ASLT), which was also surrounded by Han Chinese populations. PCA based on the top six components could explain 74.52% variance (PC1 to PC6: 34.18, 14.49, 11.70, 6.16, 5.00, and 2.99%). PC1 (Figure 1D and Supplementary Figure S2A) could distinguish the Asian populations from the others; besides, the Asian groups could be divided into two main clusters by the PC1: one contained Xinjiang and South Asian populations, and the other comprised Han Chinese, Hui, Yi, and Tibetan populations. The other five components could not separate any continental groups from the others (Supplementary Figures S3B–D).

Generally, the patterns revealed by MDS and NJ tree were in accordance with those observed in the PCA and heatmap. To directly dissect the Nagqu Tibetan ancestry component and explore the genetic similarity based on the shared ancestral components with different predefined *K* values, we conducted the STRUCTURE analysis assuming 2–6 predefined clusters (Supplementary Figure S4). We found that the fitted model with three clusters had the optimal *K* value. At *K* = 2, we identified two distinct components maximized, respectively, in ST and AT populations. At *K* = 3, population substructures of Han Chinese and Tibeto-Burman (TB) populations were observed within ST populations. Geographically, different components within the same language family gradually appeared with the increase of *K* values and the proportions of shared components were variable within ethnically different groups. Nagqu Tibetan consistently harbored a unique component and showed a closer genetic affinity with Chengdu Tibetan and Lhasa Tibetan.

### ADMIXTURE, *f_3_*-Statistics, and Phylogeny Reconstruction Among Highlanders, Eurasian Modern/Ancient References Based on the 1240K SNPs

**Figure 2:**
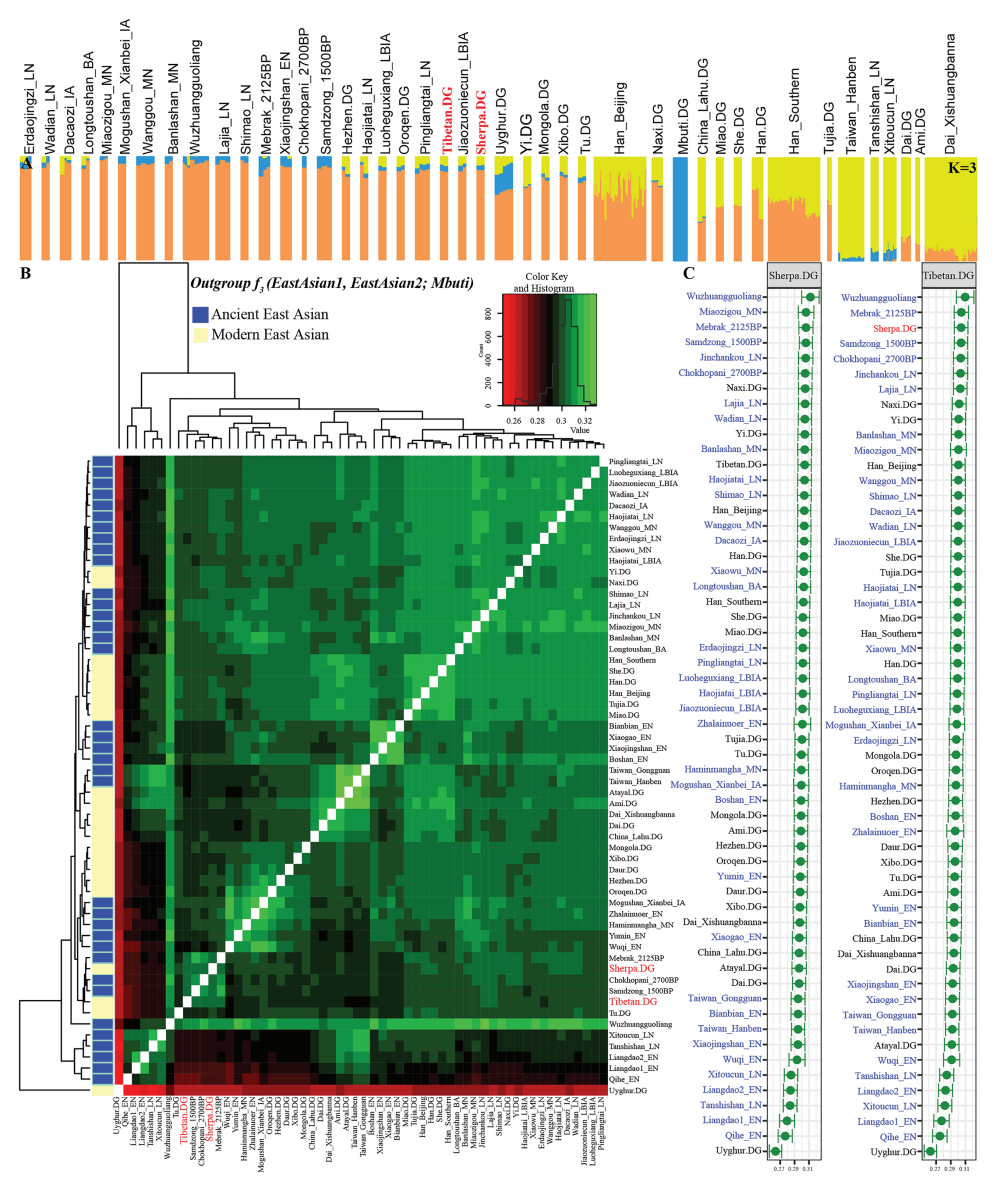
Patterns of genetic structure between Highlanders of Tibetan and Sherpa and modern/ancient East Asians. **(A)** ADMIXTURE results showed individual genetic similarity with the optimal *K* value of 3. **(B)** Heatmap showed the pairwise genetic distance among Tibetan, Sherpa, and East Asian reference populations. **(C)** The shared genetic drift between Sherpa or Tibetan and their reference populations estimated *via* outgroup-*f_3_*(Reference populations, Tibetan/Sherpa; Mbuti).

To study the demographic history and deep population history of East Asian Highlanders of Tibetan and Sherpa, we used the Tibetan and Sherpa individuals included in the Simons Genome Diversity Project (SGDP) as the new studied subject. We merged them with other publicly available modern and ancient Eurasian genomes based on the 1240K overlapping SNPs. The final dataset included 44 populations: 356 modern individuals from 21 East Asian groups and 1 central African Mbuti and 112 Chinese ancients from 22 spatiotemporally diverse archeological sites (Supplementary Figure S1). We pruned 335,589 linked SNPs from 1,233,013 SNPs and remained 897,424 markers for model-based ancestry sources modeling. Model-based ADMIXTURE results also showed the population similarities with different predefined genetic clusters. Individual and average population cluster-specific compositions are presented in Figure 2A; Supplementary Figures S5, S6; Supplementary Table S7. The fitted model with two predefined clusters separated Mbuti from other East Asians. The optimal cluster sources could be modeled and obtained when the three predefined genetic clusters (*K* = 3) were assumed (cross-validation error = 0.8832). Also, this three-population model showed that yellow ancestry was enriched in Taiwan Iron Age population (average proportion is 0.954 as blue component in Supplementary Figure S7), which also existed with a higher proportion (larger than 0.772) in the coastal late Neolithic southern East Asians (Tanshishan_LN and Xitoucun_LN) and modern southern Chinese Austronesian (Ami) and Tai-Kadai speakers (Xishuangbanna Dai). The other East Asian-dominant component (orange in Figure 2A, *K* = 3) maximized in the inland middle Neolithic northern East Asian Miaozigou individuals associated with Miaozigou culture (0.983), followed by the late Neolithic Shimao and Neolithic Wuzhuangguoliang people in Shaanxi and other ancient Tibetan and northern Chinese ancients (larger than 0.869). Modern Tibetan harbored 0.866 Miaozigou-related component and others from Hanben- or Mbuti-like component, and Sherpa derived 0.878 of their components related to this group. We identified two southern East Asian components when the model of four predefined cluster sources was used: island/coastal southern East Asian components maximized in Taiwan Iron Age Hanben people (0.962) and late Neolithic Xitoucun and Tanshishan (0.741 and 0.713, respectively) and inland southern East Asian component enriched in Tai-Kadai Dai which also existed with a high proportion in Chinese southern Tibeto-Burman Lahu, modern Austronesian Ami. and Hmong-Mien Miao and She. Similar to the patterns in *K* = 3, the third component was maximized in the inland northern Neolithic people. Based on the shared component in Figure 2A, modern Tibetan shared more components with Highland Sherpa.

We subsequently estimated the shared genetic drift between the highland East Asians (Tibetan and Sherpa) and other 350 lowland modern East Asian individuals from 20 populations, 118 lowland ancient East Asians from 33 populations, and 8 highland East Asian individuals from 3 Nepal populations (2,125-year-old Mebrak, 1,500-year-old Samdzong, and 2,700-year-old Chokhopani) *via* the outgroup *f_3_*-statistics in the form of *f_3_(Reference populations, Tibetan/Sherpa; Mbuti)*. Pairwise-shared genetic drift among 63 ancient and 22 modern East Asian populations were also calculated *via f_3_(Reference ancient/modern populations1, Reference ancient/modern populations2; Mbuti)* and submitted in Supplementary Table S7. The observed larger *f_3_* values or green color in Figure 2B denoted more shared ancestry among two reference populations, and smaller *f_3_* values or red color meant less shared ancestry among them. The red color with Uyghur and the green color with the late Neolithic Wuzhuangguoliang people observed in the heatmap, respectively, showed their genomic differentiation and similarities with reference East Asians. The cluster patterns in the heatmap showed that Tibetan clustered with Nepal ancients and kept a close relationship with Sherpa. Focused on the genetic variations of Sherpa and Tibetan (Figure 2C), we found that the top shared ancestry with highland Tibetan and Sherpa was provided by Shaanxi Wuzhuangguoliang Neolithic people (0.3096 with Tibetan and 0.3121 with Sherpa). The indexes between Tibetan and four high-altitude populations (three Nepal ancients and one modern Sherpa) were larger than 0.3034, followed by late Neolithic Qijia people from the upper Yellow River basin (Jinchankou and Lajia) and modern lowland Tibeto-Burman-speaking Naxi and Yi and other northern modern and ancient populations. Consistent patterns of genetic affinity were observed in the relationship between Sherpa and other East Asian-associated reference populations.

We subsequently estimated admixture signals of Highland East Asians *via* admixture *f_3_*-statistics in the form of *f_3_(Source population1, Source population2; targeted populations of Tibetan/Sherpa)*. The observed statistically significant negative *f_3_* values with absolute *Z* scores larger than three indicated that the targeted investigated population was a mixed population with the possible ancestral populations related to the two used sources. No negative *f_3_* values were identified in *f_3_(Source population1, Source population2; Sherpa)* among 1,653 pairs of modern and ancient East Asians, but eight population pairs with negative *f_3_* values were observed in *f_3_(Source population1, Source population2; Tibetan)* with one source from Nepal ancients and the other from modern/ancient northern East Asians (Supplementary Tables S8, S9). We should be cautious that the observed negative *f_3_* values with *Z* scores were larger than negative three. Thus, compared with obvious admixture signatures from northern and southern East Asians observed in the lowland East Asians, the highland Tibetan and Sherpa showed their unique genetic structure, which is different from other lowland East Asians.

**Figure 3:**
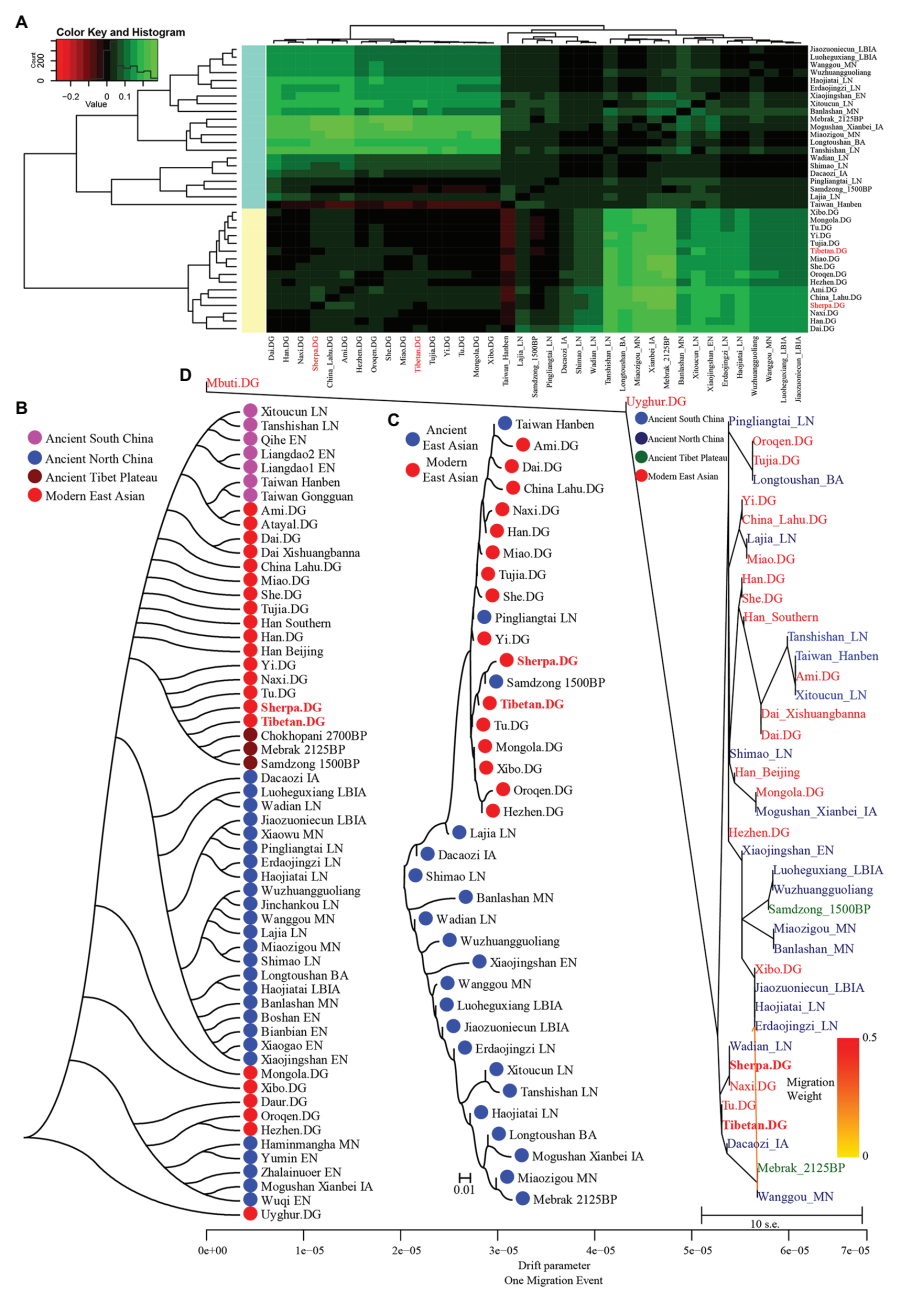
Clustered patterns among East Asian Highlanders and other reference populations. **(A)** Heatmap of the pairwise Fst genetic distances among 42 included East Asian populations. **(B)** Neighbor-joining (NJ) tree was constructed using 1 − outgroup *f_3_(Source1 Source2; Mbuti)*. **(C)** The NJ tree was constructed *via* the Fst genetic distance matrixes. **(D)** The maximum likelihood tree showed the patterns of population splits and genetic admixture with one migration event.

We also calculated Wright’s fixation index Fst among 42 modern and ancient populations (Supplementary Table S10), with the exception of the unexpected Fst values caused by the unbalanced sample size; Tibetan possessed the smallest genetic distance with Sherpa (0.0173), followed by Tu (0.0195), late Neolithic Pingliangtai (0.0236), Yi (0.0272), and Naxi (0.0289). However, Tibetan showed a close genetic relationship first with Chinese lowland Tibeto-Burman-speaking populations and then with Sherpa, which showed the more genetic influence or closer links between Tibetan and Lowland East Asian populations. Our Fst-based heatmap in Figure 3A revealed that modern and ancient populations showed their close genetic relationship within themselves. To further explore the phylogenetic relationships between Highlanders and lowland East Asians, we reconstructed three different phylogeny trees (Figures 3B–D). The first tree shown in Figure 3B was constructed using the genetic matrix of one minus outgroup*-f_3_* values (1 − *f_3_*) and NJ algorithm. Here, we could identify southern Neolithic to Iron Age populations grouped together and then grouped with southern Chinese modern Austronesian, Tai-Kadai, Hmong-Mien, and Sinitic language speakers, which formed the southern East Asian branch. Hanben and Gongguan people from Taiwan kept the closest relationship with modern Austronesian Ami and Atayal. Sherpa and Tibetan possessed a strong genetic affinity and grouped first with three Nepal ancients and then with lowland Tibeto-Burman-speaking Tu, Naxi, and Yi and formed the TP branch. The observed Tibeto-Burman branch showed a close genetic relationship between modern lowland/highland Tibeto-Burman language speakers and ancient highland Nepal ancients. The northern ancient branch comprised early Neolithic to Iron Age individuals from Shandong and Henan provinces in the middle and lower Yellow River basin and from Shaanxi and Qinghai provinces in the upper Yellow River basin and West Liao River. Amur River ancient clustered with modern Tungusic and Mongolic speakers formed an Amur branch. The overall patterns observed in the *f_3_*-based phylogenetic relationship showed the TP branch was placed in the intermediate position between the northern East Asian branch and the southern East Asian branch, but far away from the Amur branch. The second NJ tree based on the Fst genetic distance matrices clustered one modern population branch and one ancient population branch (Figure 3C). Although there was separation between modern and ancient populations in the clustered results, we could also identify that Hanben was grouped with modern Ami, late Neolithic Pingliangtai clustered with Yi and She, and the studied Tibetan and Sherpa Highlanders grouped with 1,500-year-old Samdzong. In the third one, we considered the gene flow events among the patterns of population splits and admixture among East Asians and reconstrued one maximized likelihood tree (Figure 3D). We found highland Tibetan and Sherpa grouped with their geographically/linguistically close populations. Similar clustered patterns were identified among southern modern and ancient East Asians and northern modern and ancient East Asians. No obvious gene flow events into Tibetans or from Tibetans into other East Asians were identified.

### Genomic Affinity and Differentiation Between Sherpa and Tibetan Revealed by *f_4_*-Statistics

**Figure 4:**
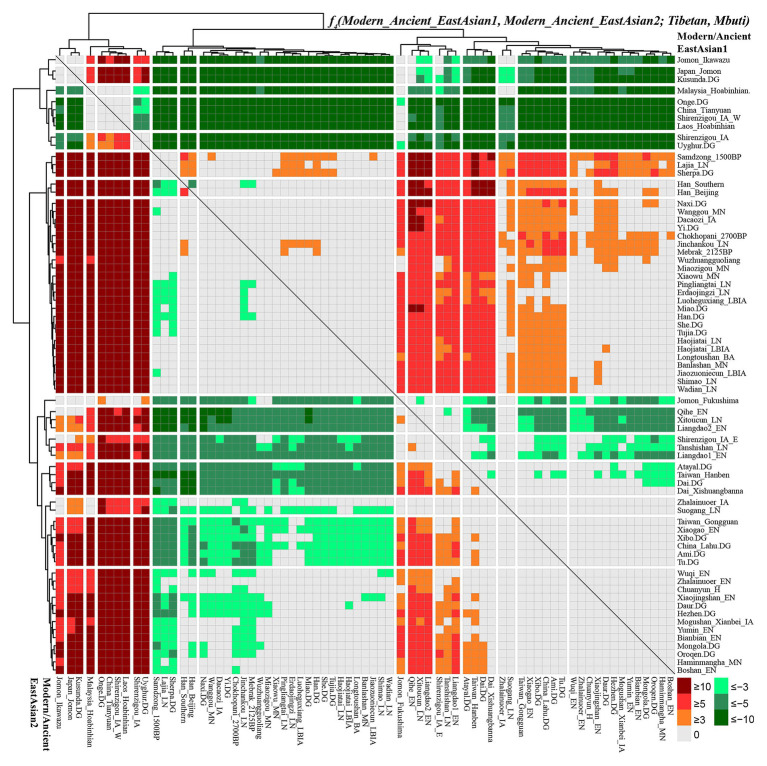
Shared genetic drift between highland East Asian Tibetan and other modern and ancient reference populations *via f_4_(modern/ancient East Asian1, modern/ancient East Asian2; Tibetan, Mbuti)*.

To comprehensively evaluate the genetic relationships between highland Tibetan and Sherpa, we performed four-population comparisons (*f_4_*-statistics) to explore the differentiated shared drifts between Highlanders and lowland East Asian reference groups compared with other East Asian reference groups in the form of *f_4_(Modern/Ancient Chinese population1, Modern/Ancient Chinese population2; Tibetan/Sherpa, Mbuti)*. The observed significant negative *f_4_* values with the absolute *Z* scores larger than three (green color in the heatmap) denoted that our studied Tibetan and Sherpa shared more genetic drifts with Modern/Ancient Chinese population2 relative to the Modern/Ancient Chinese population1; otherwise, significant positive *f_4_* values (red color in the heatmap) denoted more shared alleles between Highlanders and Modern/Ancient Chinese population1 rather than Modern/Ancient Chinese population2. No significant negative or positive *f_4_* values (*Z* scores ranging from −3 to 3, gray color) denoted two Chinese reference populations formed one clade relative to our studied Highlanders. As shown in Supplementary Table S11 and Figure 4, *f_4_(Xinjiang ancient/modern populations, other modern/ancient East Asians; Tibetan, Mbuti)* was conducted to explore the relationships between Highlanders and northwestern Chinese populations (modern Uyghur and Iron Age Shirenzigou people). The results of significant negative *f_4_* values showed that Tibetan shared more derived alleles with both northern and southern Neolithic to present-day East Asians than with Xinjiang Iron Age to modern populations, which suggested little genetic materials associated with western Eurasian in Tibetans (Ning et al., 2019). Compared with 40,000-year-old Tianyuan people, Tibetan shared more alleles with modern Uyghur [*f_4_*: 3.546*standard error (SE)], Shirenzigou_IA (4.624*SE), and Shirenzigou_IA_E (8.593*SE) *via f_4_(Xinjiang populations, China_Tianyuan; Tibetan, Mbuti)*. Compared with Shirenzigou individuals with stronger western Eurasian affinity, we found that Tibetan shared more alleles with modern Uyghur, Shirenzigou_IA, and Shirenzigou_IA_E. Moreover, genetic similarities between Tibetan and Shirenzigou_IA_E were further confirmed *via f_4_(Shirenzigou_IA_E, Shirenzigou_IA/Uyghur; Tibetan, Mbuti) =* 5.827*SE/9.473*SE. To study the genetic links between Tibetans and early East Asians, we carried out *f_4_(Early Asians, modern/ancient East Asians; Tibetan, Mbuti)* and found that Tibetan shared more derived alleles with Neolithic to present-day East Asians compared with deep East Asian lineages. Here, early East Asians were represented by Onge from South Asia, Hoabinhian people from Laos and Malaysia, and Tianyuan from Beijing. Compared with early East Asians, Tibetan shared more alleles with modern and ancient East Asians with negative *f_4_* values in the *f_4_(Early East Asians, modern/ancient Chinese populations; Tibetan, Mbuti)*. Some cases with the more shared genetic drifts between Tibetan and Jomon people were identified when we used Xinjiang Iron Age to modern groups or 40,000-year-old Tianyuan people as the reference populations, such as *f_4_(Ikawazu Jomon, Shirenzigou_IA; Tibetan, Mbuti) =* 5.529*SE. In summary, compared with northwestern Chinese populations with signatures of western Eurasian admixture and early East Asians, we found a strong genomic affinity between our studied Tibetan and northern/southern lowland East Asians. These observed genetic close relationships between Highlanders and East Asians showed that the gene pool of modern Tibeto-Burman speakers mainly originated from East Asians, not from South Asia or Central Asia, although too many natural corridors and historic or prehistoric trade routes connected the TP and Central Asia or the Indian subcontinent (Jeong et al., 2016).

Focused on the population substructure within East Asians, the negative *f_4_* values in *f_4_(coastal Neolithic southern East Asians, modern/ancient northern East Asian; Tibetan, Mbuti)* showed that Tibetan shared more alleles with northern East Asians. Positive values in *f_4_(Hanben/Atayal, coastal Neolithic southern East Asians; Tibetan, Mbuti)* suggested Tibetan shared more alleles with Iron Age Hanben Taiwanese and their descendants than with their ancestors (Early Neolithic people), and positive *f_4_* values in *f_4_(inland modern southern East Asian Dai, coastal Neolithic to present-day southern East Asians; Tibetan, Mbuti)* denoted Tibetan shared more alleles with inland southern East Asians than with island/coastal southern East Asians. Consistent positive *f_4_* values in *f_4_(Samdzong_1500BP/Lajia_LN/Sherpa, Lowland East Asians; Tibetan, Mbuti)* showed that Tibetan had a strong genetic affinity with 1,500-year-old Samdzong people and Qijia people from Lajia, as well as the modern Sherpa. This obvious genetic affinity between modern Tibetans and ancients from Nepal and Qinghai showed the direct genetic contribution between Qijia culture-associated ancestral population and modern Tibetan or Nepal high-altitude people and modern Highlanders. Affinity between Tibetan and modern Tibeto-Burman speakers and other northern East Asians was further confirmed *via* positive *f_4_* values in *f_4_(northern East Asians, southern East Asians/Mongolic/Tungusic speakers; Tibetan, Mbuti)*. Focused on the Sherpa, as shown in Supplementary Figure S8 and Supplementary Table S12, all green color denoted the significant negative *f_4_* values in *f_4_(modern/ancient East Asian1, modern/ancient East Asian2; Sherpa, Mbuti)*, which suggested Sherpa shared more derived alleles with lowland and highland northern East Asians compared with the early Asians, northwestern Chinese populations with western Eurasian admixture, ancients from coastal southeast China, islanders of Taiwan and Japanese Archipelago, and even some southern Chinese indigenous populations of Atayal and Dai. Red colors were observed when we used the following groups as the Modern/Ancient East Asian1: middle Neolithic populations (Miaozigou_MN, Wanggou_MN, Banlashan_MN, Wuzhuangguoliang), late Neolithic people (Wadian_LN, Haojiatai_LN, Shimao_LN), Qijia people (Jinchankou_LN, Lajia_LN, Dacaozi_IA), ancient Tibetans (Chokhopani_2700BP, Mebrak_2125BP, Samdzong_1500BP), and modern Sino-Tibetan (Naxi, Yi, Tibetan, Han, Han_Southern, Han_Beijing), which showed that Sherpa shared more alleles with them compared with southern East Asians or early Neolithic northern East Asians.

**Figure 5:**
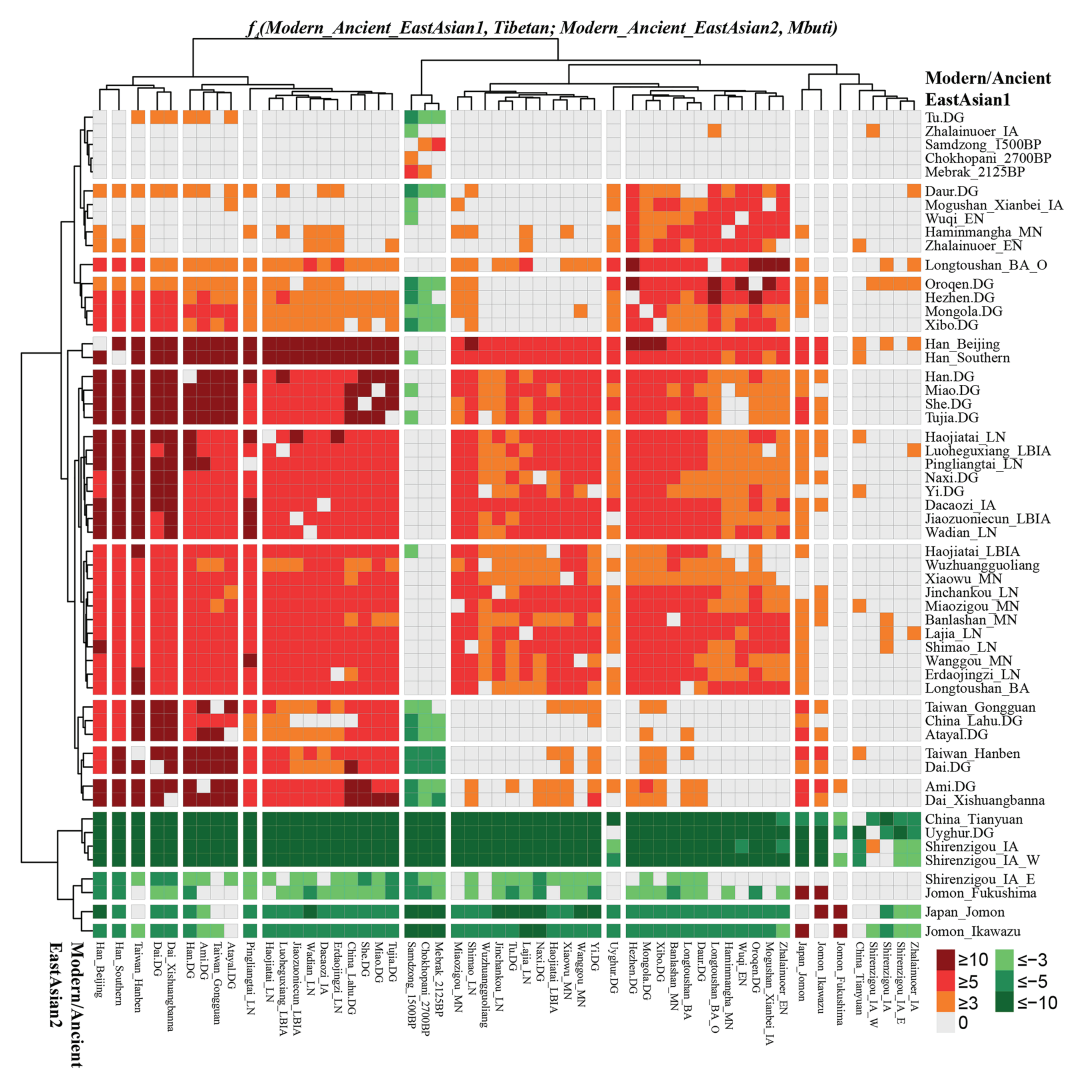
Results of *f_4_(modern/ancient East Asian1, Tibetan; modern/ancient East Asian2; Mbuti)* showed more or less modern/ancient East Asian2-related ancestry in Tibetan relative to modern/ancient East Asian1.

To further explore the genetic continuity and admixture of the Highlanders of Tibetan and Sherpa, we performed affinity *f_4_* statistics in the form of *f_4_(modern/ancient East Asian1, Tibetan/Sherpa; modern/ancient East Asian2, Mbuti)*. As shown in Figure 5 and Supplementary Table S13, population lists of modern/ancient East Asian1 were presented in the right part and the other one was listed in the bottom part. Green colors showed the negative *f_4_* values, which suggested that Tibetan harbored more ancestry derived from the groups related to the modern/ancient East Asian2. Here, we found that Tibetan possessed more ancestry from both northern and southern modern/ancient East Asians compared with northwestern Chinese populations and early Asians (Tianyuan, Hoabinhian, and Jomon). Most negative *f_4_* values were also observed in *f_4_(Qihe_EN/Liangdao2_EN, Tibetan; northern East Asians, Mbuti)*, suggesting that Tibetans harbored more northern East Asian ancestry. Red colors showed strong genetic affinity among lowland East Asians or more shared ancestry among them relative to Tibetan. We expected to observe the significant negative *f_4_* values if the included modern/ancient East Asian2 was the direct ancestor of Tibetan. Interestingly, *f_4_(modern/ancient East Asian1, Tibetan; Samdzong_1500BP/Sherpa/Mebrak_2125BP/Chokhopani_2700B, Mbuti)* showed negative *f_4_* statistical values. However, no similar signals were identified in the late Neolithic Lajia or Jinchankou populations. Furthermore, no significant *f_4_* values should be observed when Nepal ancients were the unique ancestral source, or negative *f_4_* values could be obtained if there were some additional admixture gene flow into modern Tibetan in *f_4_(Samdzong_1500BP/Sherpa/Mebrak_2125BP/Chokhopani_2700B, Tibetan; modern/ancient East Asian2, Mbuti)*. No statistically significant *f_4_* values were observed here suggesting that ancestral populations related to the Nepal ancients were the direct ancestors of modern Tibetans. Although differentiated shared alleles were observed between Highlanders of Tibetan and Sherpa illustrated *via f_4_(Tibetan, Sherpa; Tu/Atayal/Niaozigou_MN/Wadian_LN/Erdaojingzi_LN, Mbuti)*, similar patterns of shared genetic drift were identified in Sherpa populations (Supplementary Figure S9 and Supplementary Table S14).

### Genetic Admixture History Reconstruction of Highlanders of Tibetan and Sherpa *via qpWave/qpAdm* and *qpGraph*

**Figure 6:**
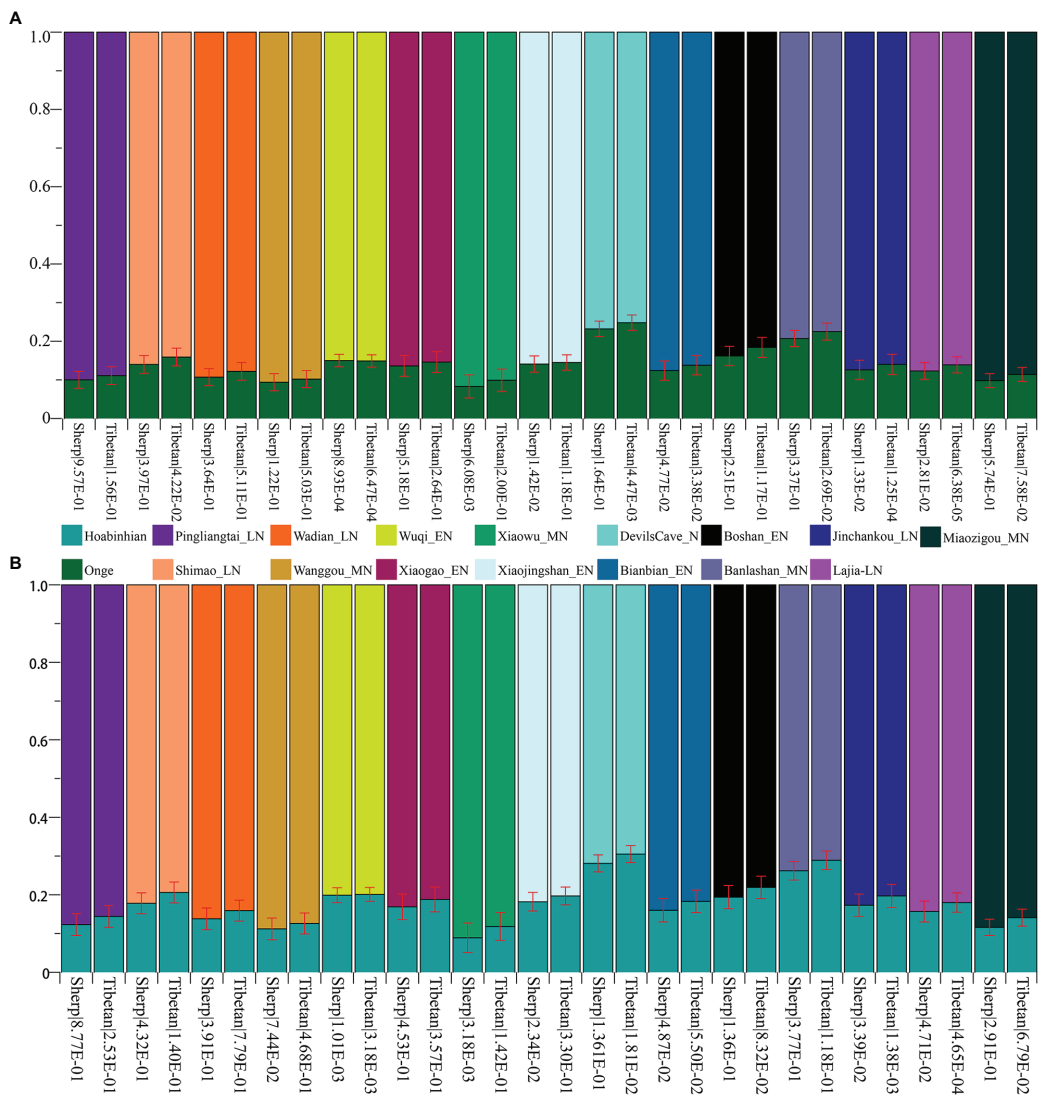
Ancestry composition of East Asian Highlanders of Tibetan and Sherpa under the two-way admixture model. Yellow River farmers were used as one Neolithic ancestry sources and early Asians of modern Onge **(A)** and ancient Hoabinhian **(B)** as the other source.

Subsequently, to explore the plausible models of admixture fitted well of Highlanders and estimate the corresponding ancestry proportion, we used the statistical tool of *qpWave* to explore the minimum number of the possible ancestral populations and *qpAdm* to qualify ancestry proportion. Eight populations (Mbuti, Russia_Ust_Ishim, Russia_Kostenki14, Papuan, Australian, Mixe, Russia_MA1_HG, Mongolia_N_East) were used as the base outgroup set. Our *qpWave* results of p_rank0 <0.05 showed at least two ancestral populations could be used to model the ancestry composition of our included Tibetan and Sherpa. We first used the Ancient Ancestral South Indian of Onge as the southern source population represented as the deep diverged eastern Eurasian ancestry, which recently was hypothesized as the representation of indigenous South Asians in the study of the formation of human populations in South Asia (Narasimhan et al., 2019). Fifteen Neolithic northern East Asians from the Yellow River basin, West Liao River basin, Amur River basin, and other northern China and Russia were used as the other northern ancestral source. As shown in Figure 6 and Supplementary Table S15, six models could be used to fit the observed genetic variations in both Sherpa and Tibetan with a large proportion of northern East Asian ancestry and small highly diverged eastern Eurasian ancestry: included two coastal early Neolithic northern East Asian models (Boshan_EN and Xiaogao_EN), two inland middle Neolithic northern East Asian models (Miaozigou_MN and Wanggou_MN), and two inland late Neolithic northern East Asian models (Wadian_LN and Pingliangtai_LN). Besides, we also found Sherpa could be modeled as the admixture of 0.860 ± 0.023 Shimao-related ancestry and 0.140 Andamanese hunter-gatherer-related ancestry, 0.768 ± 0.02 DevilsCave_N-related ancestry and 0.232 Onge-related ancestry, and 0.793 ± 0.021 Banlashan Hongshan people-related ancestry and 0.207 Onge-related ancestry. Similarly, Tibetan could be modeled as the mixing of 0.855 ±0.020 Xiaojingshan_EN-related ancestry and 0.145 Onge-related ancestry, or 0.901 Xiaowu_MN-related ancestry and 0.099 Onge-related ancestry. Sherpa people could be modeled as approximately 0.870 of their ancestry derived from Qijia people associated with Lajia and Jinchankou populations with marginal nonsignificant *p* values (1.33E-02 and 2.81E-02). When we substituted Hoabinhian from Laos with Onge as the southern ancestral source, the aforementioned six models (two early Neolithic sources of Xiaogao and Boshan, two middle Neolithic sources of Wanggou and Miaozigou, and two late Neolithic sources of Pingliangtai and Wadian) could be fitted well of two included Highlanders with relative higher ancestry proportion from Hoabinhian-related ancestry. Another three models (Bianbian-EN-Hoabinhian: 0.840 for Sherpa and 0.817 for Tibetan; Banlashan_MN-Hoabinhian: 0.738 for Sherpa and 0.711 for Tibetan; and Shimao_LN-Hoabinhian: 0.877 for Sherpa and 0.856 for Tibetan). Middle Neolithic Xiaowu (0.882) and Xiaojingshan_EN (0.818) could be used as the northern East Asian sources for the model of the formation of modern Tibetan, and DevilsCave_N could be used as the source for modeling modern Sherpa with 0.719 derived from northern sources.

**Figure 7:**
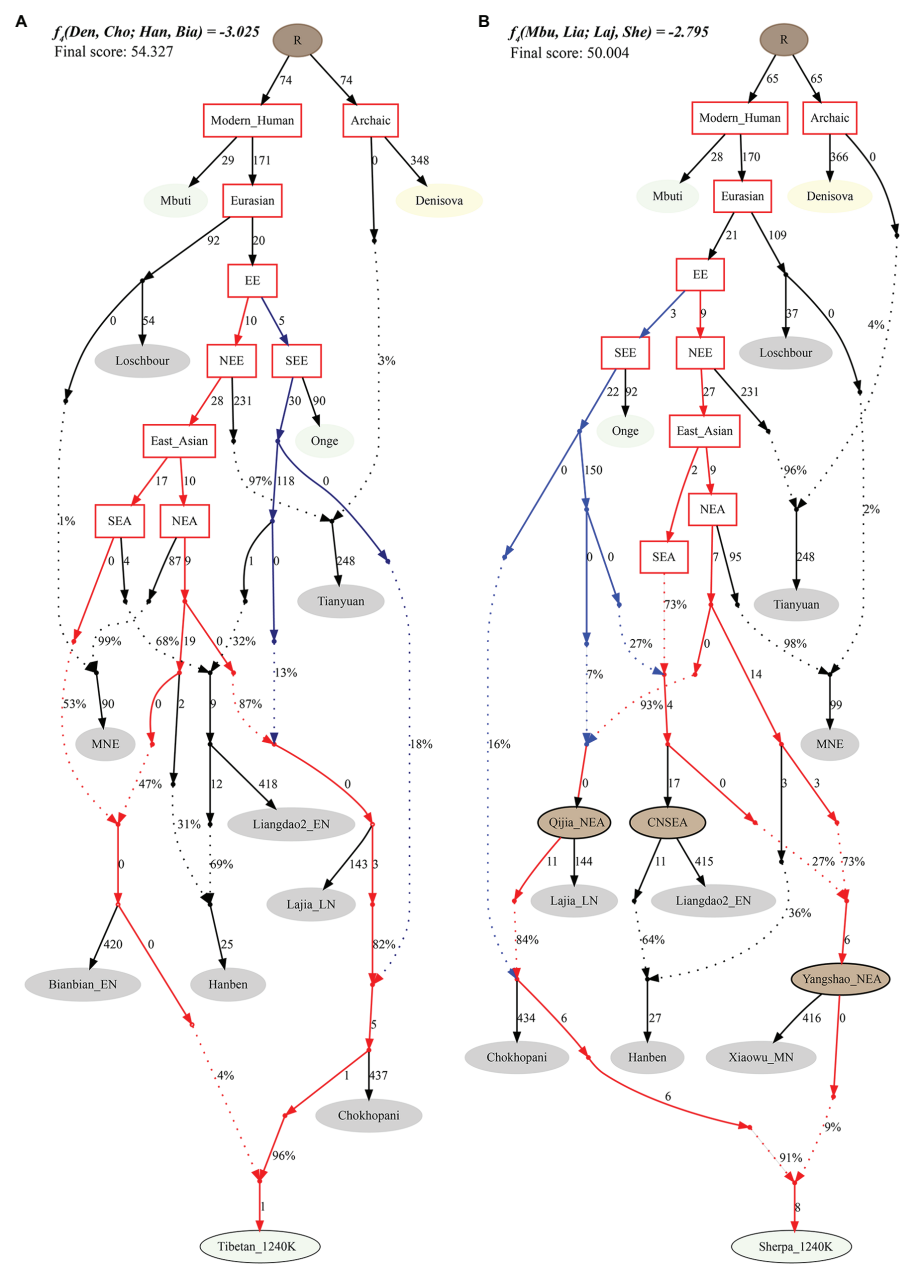
Phylogenetic framework of Tibetan **(A)** and Sherpa **(B)** based on the 1240K high-density datasets.

Finally, to reconstruct a deep population admixture history of the Highlanders of Tibetan and Sherpa based on the 1,233,013 SNPs, we used the basic phylogenetic framework from Wang et al. with the terminal modern populations of Mbuti, Onge, archaic population of Denisovan, and Paleolithic to Iron Age populations of Loschbour, Tianyuan, Liangdao2_EN, Lajia_LN, Chokhopani, and two eastern Mongolia Neolithic people (Wang et al., 2020). After adding Tibetan and Sherpa populations from the Simons Genome Diversity Project, we found Tibetan could be modeled as mixing from three source populations (Figure 7): coastal early Neolithic northern East Asian Bianbian_EN-related (Houli people: 0.040), inland late Neolithic northern East Asian Lajia_LN-related (Qijia people: 0.787), and deeply diverged East Eurasian-related (first layer of indigenous people, 0.173). For Sherpa, we used the middle Neolithic Yangshao people (Xiaowu_MN-related) as one of the northern East Asian sources, which could be modeled as the admixture of 0.73 ancestry directly derived from the northern main ancestral lineage and obtained additional 0.27 ancestry from southern East Asian lineage. In this situation, Sherpa was modeled as 0.09 ancestry from a group related to the middle Neolithic Yangshao people, 0.7644 from the ancestral population related to Lajia_LN, and 0.1456 from deeply diverged eastern Eurasian.

## Discussion

The Qinghai-Tibet Plateau and the surrounding great mountain ranges are home to cultural, genetic, and linguistic diversity since prehistoric or historic times, although nature environments, such as high-altitude hypoxia, resource scarcity, cold stress, and rough terrain, to some extent hindered the process, scale, and speed of the population’s settlement in this world’s high plateau. Archeological documents from Xiahe Denisovan mandible in northeastern TP (3,280 m above sea level) and abundant blade tool assemblage in the Nwya Devu site (4,600 m above sea level) successively demonstrated that humans colonized this high-altitude area from late Middle Pleistocene (160,000 years ago) to late Paleolithic stage (40,000–30,000 years ago; Zhang et al., 2018; Chen et al., 2019a). Genetic evidence for the high-altitude adaptative Denisovan-derived EPAS1 haplotype observed in modern Tibetan further showed a partial genetic continuity or archaic introgression between Denisovan and modern East Asian Highlanders. However, the demographic history and fine-scale genetic structure of modern and ancient Highlanders kept unclear and needed to be comprehensively explored. In the present study, we first used forensic short tandem repeat markers with high polymorphic and informative features to explore the genetic relationships between highland Tibetan and worldwide reference populations based on the allele frequency spectrum and found that East Asian Highlanders had a close genetic relationship with modern Tibeto-Burman-speaking populations and northern Han Chinese. This observed pattern of population relationship based on low-density genetic markers was consistent with recent linguistic evidence for the North China origin of modern Sino-Tibetan language (Sagart et al., 2019; Zhang et al., 2019).

To further clarify the population relationship and potential gene flow events, we subsequently used one high-density dataset comprised of the 1240K SNP genetic markers focused on the Highlanders of Tibetan and Sherpa and compared them with all available Chinese ancient and modern reference populations (Patterson et al., 2012; Yang et al., 2017, 2020; Lipson et al., 2018; Jeong et al., 2019; Liu et al., 2020; Ning et al., 2020; Wang et al., 2020) to carry out another comprehensive population genetic relationship analysis. Ancestry composition *via* the ADMIXTURE model-based cluster result showed a genetic affinity between Tibetan and Sherpa and their close genetic relationship with eight Nepal ancient individuals from a cultural background associated with Chokhopani, Mebrak, and Samdzong. This observed genetic similarity and continuity based on the 1240K dataset were consistent with Jeong’s original finding of long-term genetic stability (Jeong et al., 2016). Genetic affinity and continuity among ancient Nepal populations and modern Tibetan and Sherpa were further evidenced *via* the more shared genetic drift in *f*-statistics and close phylogenetic relationships in the NJ tree and *qpGraph*-based phylogeny framework. Besides, we also identified a close genetic relationship between modern Sherpa/Tibetan and ancient Qijia people from the upper Yellow River basin (Lajia and Jinchankou), suggesting Qijia people as the representative of Neolithic millet farmers played an important role in the formation of modern Tibetans although they shared more alleles with Neolithic Yangshao, Longshan people from Central Plain in Henan Province, and Houli people from Shandong Province. Our autosome-based genetic links between ancient populations from northeast TP were consistent with recent archeological, Y-chromosomal, and mitochondrial evidence for the colonization and peopling of the Qinghai-Tibet Plateau (Chen et al., 2015; Wang et al., 2018a; Zhang et al., 2018; Li et al., 2019b; Ding et al., 2020). Archeologically attested charred grains and the corresponding carbonization dating data provided by Chen et al. suggested that a novel agropastoral economy facilitated Neolithic millet farmers to enjoy year-round living and to successfully occupy the northeastern TP around 3,600 years ago (Chen et al., 2015). Mitochondrial DNA (mtDNA) variations of modern Tibetan also provided clues that the upper Yellow River millet farmers first adopted cold-tolerant barley agriculture and then permanently inhibited it in the TP (Li et al., 2019b). Ancient mitogenomes of 5,200- to 300-year-old humans from Tibet, Gansu, Qinghai and Sichuan provinces also revealed that the D4j1b-represented ancestral population expanded from the low-altitude area to the core region of the TP around 4,750 to 2,775 years ago (Ding et al., 2020). Uniparental genetic evidence from Y-chromosome phylogeny also showed that the Yellow River farmers with the paternal founding lineage of O*α*1c1b-CTS5308 dispersed to the TP had triggered the formation and expansion of modern high-altitude Tibeto-Burman speakers (Wang et al., 2018a). Thus, our findings combined with evidence from the aforementioned archeological or uniparental contents consistently supported that the northeastern edge of the TP is an important geographical corridor for ancient human movements and admixtures between low altitude and high altitude.

Both southwestern agricultural charred cereal grains (barley and wheat) and northern China Yellow River dryland millet charred cereal grains (foxtail millet and broomcorn millet) were identified in the Neolithic archeological sites in the northeastern TP (Chen et al., 2015), suggesting that the communication of the adaptation of agriculture techniques existed there. A close genetic connection combined with these archeological records evidenced that the northeastern TP is the main geographical corridors of the peopling of TP. However, whether this mixed agriculture system was caused by human population movements and admixture or only acculturation of skills is unclear. We performed a series of population genetic analyses to clarify the admixture sources and progress. First, *f_3_* and *f_4_*-*statistics* did not identify more shared genetic drift with western Eurasian populations. Second, the observed genetic variations observed in Highlanders of Sherpa and Tibetan could be competently explained *via* a two-way admixture model with one deeply diverged Onge/Hoabinhian-related eastern Eurasian lineage and one northern East Asian lineage. Third, our *qpGraph*-based admixture graph model fitted well without a gene flow from western Eurasian populations. Thus, our genetic phylogenetic evidence supported that the upper Yellow River millet farmers adopted western barley and wheat agriculture techniques *via* an adaptation of the idea and not the direct movement of people. The cultural diffusion model was recently also evidenced *via* mitochondrial haplotype and haplogroup data (Li et al., 2019b). Li et al. recently discorded the founder maternal lineages (M9a1a1c1b1a and A11a1a) of Neolithic millet farmers based on the combined analyses of radiocarbon dating of cereal remains and mtDNA-based haplogroup geographical distribution among 8,277 Tibetans and 58,514 individuals from surrounding populations. Their founding supported that Yellow River millet farmers adopting barley agriculture successfully colonized the East Asian high-altitude region. In summary, our admixture-*f_3_* results, symmetrical-*f_4_* analyses, and *qpGraph*-based phylogeny did not identify obvious western Eurasian-related gene flow events in Qijia people and modern Highland East Asians, which suggested that cultural communication did not involve large-scale population movements and admixtures from Central Asia or western Eurasia.

Different from subpopulation structures observed in Highland East Asians (Zhang et al., 2017), our present study identified a genetic similarity between Sherpa and Tibetan, which may be caused by the small sample size and low density of genetic sampling. Thus, denser sampling of geographically/ethnically/linguistically diverse modern highland East Asians and ancient populations should be done to clarify the population substructure and demographic history of modern and ancient highland/lowland East Asians. Regardless of the fact that the limitations of sample size and population numbers existed here, our ancestry composition estimation and phylogeny reconstruction revealed multiple stages of genetic admixtures of both Tibetan and Sherpa. Paleolithic ancestry was estimated to over 10% when we used the South Asian Onge (shared deeply diverged haplogroup D) and early Asians of Laos Hoabinhian as the deep ancestral source. This deeply diverged eastern Eurasian identified in modern East Asian Highlanders and 2,750-year-old Nepal ancient was consistent with Paleolithic sublineages of haplogroup D-M174 (D1a1-M15 and D1a2-p99), which was the representative lineage or genetic legacy of Paleolithic TP local residing hunter-gatherers (Wang et al., 2018a). This finding combined with Paleolithic archeological documents (Zhang et al., 2018), genetically attested Denisovan EPAS1 haplotype (Huerta-Sanchez et al., 2014), and Paleolithic paternal/material founding lineages (Qi et al., 2013) supported that both Paleolithic and Neolithic genetic legacies co-existed in Iron Age to modern highland East Asians.

## Conclusion

Our population genetic or genomic analyses showed that both high-density and low-density datasets in the present study revealed the close genetic relationship between Highlanders and lowland Tibeto-Burman-speaking populations, forensic-related STR-based analysis showed limitations for finer-scale genetic structure dissection due to its relatively lower resolution with the forensically developed systems. We used STR-based datasets to evaluate the genetic diversity and forensic characteristics as well as to uncover the genetic similarities and differentiation between the studied Tibetan group and 56 reference populations and found that the STR amplification system was informative and discriminative in Nagqu Tibetan and could be applied in the construction of the Chinese national STR datasets. Comprehensive worldwide or nationwide population comparisons demonstrated that Nagqu Tibetan keeps the genetic affinity with ethnically close Chengdu Tibetan, Liangshan Tibetan, and Tibet Tibetan. Furthermore, population structure and demographic history reconstruction based on the high-density 1240K dataset showed that Highlanders of Tibetan and Sherpa possessed a close genetic relationship with Qijia culture-related people (Lajia and Jinchankou), suggesting that the northeastern edge of the Tibetan Plateau is an important geographical corridor for population movements and admixtures in the progress of permanent human settlement of the TP. No western Eurasian admixture signatures were identified in modern and ancient populations of the core region and northeastern edge of the TP, suggesting that the late Neolithic upper Yellow River millet farmers’ adoption of barley and wheat agriculture from the Fertile Crescent of southwestern Asia was mediated *via* the cultural diffusion model and not *via* the demic diffusion model. Finally, the observed shared deeply diverged Onge/Hoabinhian-related eastern Eurasian lineage into modern Tibetan, Sherpa, and 2,700-year-old Chokhopani demonstrated that a common Paleolithic genetic legacy widely existed in all highland East Asians.

## Data Availability Statement

The raw data supporting the conclusions of this article will be made available by the authors, without undue reservation.

## Ethics Statement

The studies involving human participants were reviewed and approved by Ethics Committee of Zunyi Medical University. The patients/participants provided their written informed consent to participate in this study. Informed consent was obtained from all participants included in the study.

## Author Contributions

GH, MW, and XZ conceived the idea for the study. GH, PC, XZ, YL, and MW performed or supervised wet laboratory work. GH, MW, XZ, PC, ZW, JL, LY, FW, YL, and RT analyzed the data. GH, MW, YL, and XZ wrote and edited the manuscript. All authors contributed to the article and approved the submitted version.

### Conflict of Interest

The authors declare that the research was conducted in the absence of any commercial or financial relationships that could be construed as a potential conflict of interest.

## Footnotes

^1^https://www.r-project.org/
