# Supplementary material for: Combined Low-/High-Density Modern and Ancient Genome-Wide Data Document Genomic Admixture History of High-Altitude East Asians
Source: Front Genet. 2021 Feb 11;12:582357. doi: 10.3389/fgene.2021.582357 (PMC7905318; doi:10.3389/fgene.2021.582357)
Supplement: Supplementary file 1 [file Data_Sheet_1.pdf]

Combined low/high-density modern and ancient genome-wide data documents genomic admixture history of high-altitude East Asians

Yan Liu[1,*], Mengge Wang[2,*,#], Pengyu Chen[3,4,*], Zheng Wang[2], Jing Liu[2], Lilan Yao[3,4], Fei Wang[2], Renkuan Tang[5], Xing Zou[2,#], Guanglin He[2,6,#]

[1]School of Basic Medical Sciences, North Sichuan Medical College, Nanchong, Sichuan, 637100, China
[2]Institute of Forensic Medicine, West China School of Basic Science and Forensic Medicine, Sichuan University, Chengdu, China
[3]Key Laboratory of Cell Engineering in Guizhou Province, Affiliated Hospital of Zunyi Medical University, Zunyi China,
[4]Center of Forensic Expertise, Affiliated Hospital of Zunyi Medical University, Zunyi, Guizhou, China
[5]Department of Forensic Medicine, College of Basic Medicine, Chongqing Medical University, Chongqing, China
[6]Department of Anthropology and Ethnology, Institute of Anthropology, National Institute for Data Science in Health and Medicine, and School of Life Sciences, Xiamen University, Xiamen, China

*These authors contributed equally to this work and should be considered co-first authors.

#Corresponding author
Mengge Wang
Affiliation: Institute of Forensic Medicine, West China School of Basic Science and Forensic Medicine, Sichuan University, Chengdu, China
Email: Menggewang2021@163.com
Xing Zou
Affiliation: Department of Anthropology and Ethnology, Institute of Anthropology, National Institute for Institute of Forensic Medicine, West China School of Basic Science and Forensic Medicine, Sichuan University, Chengdu, China
E-mail: forensiczx@163.com
Guanglin He
Affiliation: Department of Anthropology and Ethnology, Institute of Anthropology, National Institute for Data Science in Health and Medicine, Xiamen University,
Xiamen, China.
E-mail: Guanglinhescu@163.com

Content of Supplementary Figures

Figure S1. Geographical position of included ancient East Asians used in this population genetic analysis. IA: Iron Age, W: West, E: East, EN, Early Neolithic, MN: Middle Neolithic, LN, Late Neolithic, LBIA, Late Bronze Age and Iron Age. II

Figure S2. Heatmap shows the genetic similarities and differences between Nagqu Tibetan and other 56 worldwide reference populations based on the allele frequency distributions. 1

Figure S3. Population affinity between Nagqu Tibetan and other 56 worldwide reference populations inferred from the principal component analysis based on the allele frequency distributions. 2

Figure S4. Shared composition of 3287 individuals from 11 Chinese reference populations inferred from the raw genotype data. 3

Figure S5. The distribution of cross-validation error value in model-based ADMIXTURE analysis with different predefined clusters. 3

Figure S6. Individual-level shared composition in the model-based ADMIXTURE analysis. 4

Figure S7. Population-level shared composition in the model-based ADMIXTURE analysis. 5

Figure S8. Shared genetic drift between highland East Asian Sherpa and other modern and ancient reference populations via *f_4(modern/ancient East Asian1, modern/ancient East Asian2; Sherpa, Mbuti)*. 6

Figure S9. Shared genetic drift between highland East Asian Sherpa and other modern and ancient reference populations via *f_4(modern/ancient East Asian1, Sherpa; modern/ancient East Asian2, Mbuti)*. 7

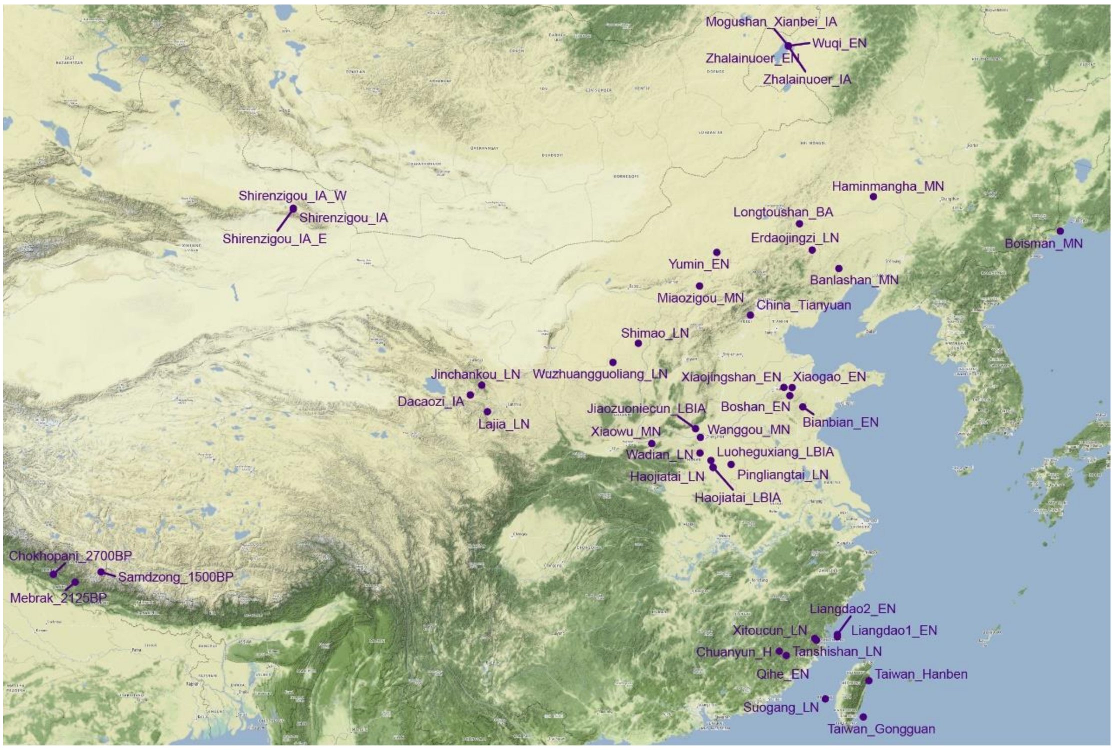

Figure S1. Geographical position of included ancient East Asians used in this population genetic analysis. IA: Iron Age, W: West, E: East, EN, Early Neolithic, MN: Middle Neolithic, LN, Late Neolithic, LBIA, Late Bronze Age and Iron Age.

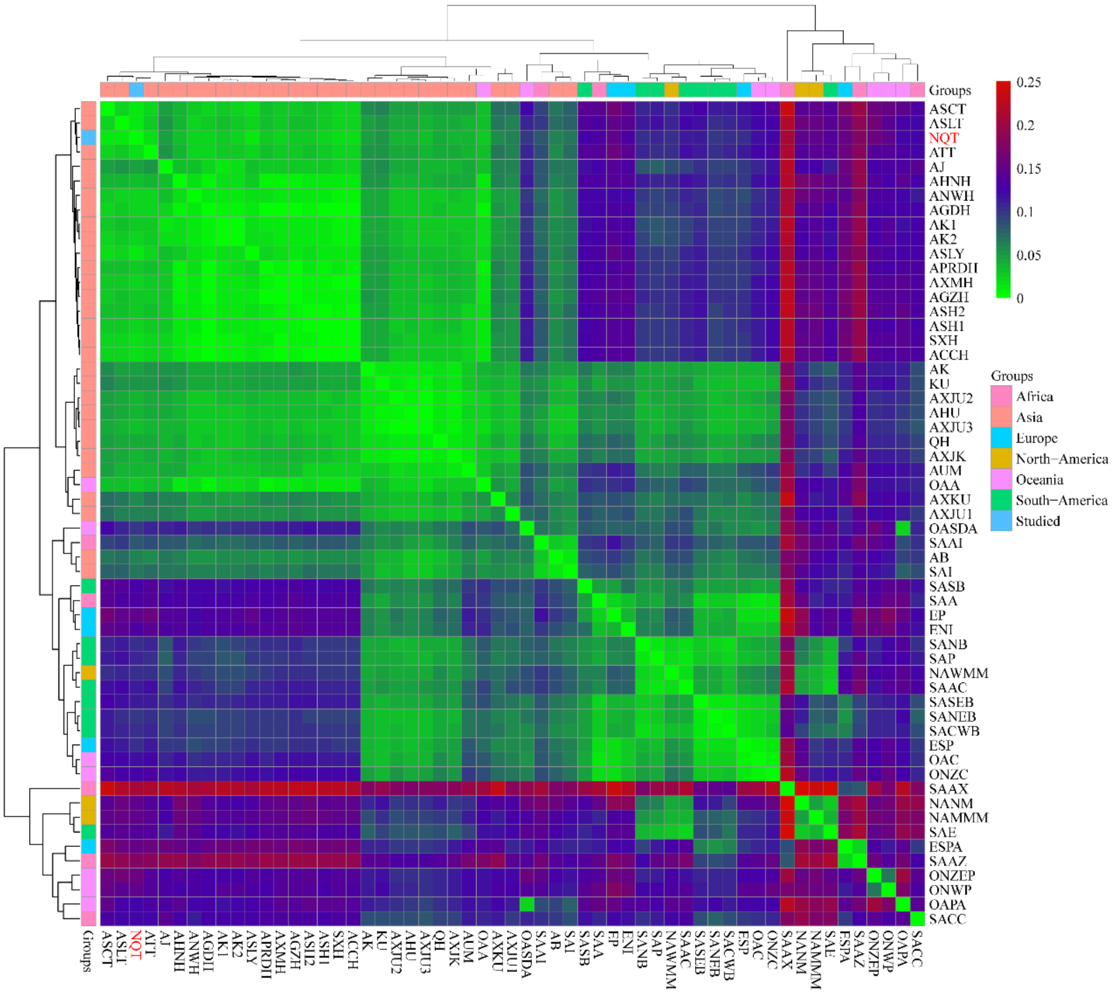

Figure S2. Heatmap shows the genetic similarities and differences between Nagqu Tibetan and other 56 worldwide reference populations based on the allele frequency distributions.

The full population names are submitted in the Supplementary Table S1. The Chengdu Tibetan (ASCT) is identified as the genetically closest population to Nagqu Tibetan (0.012), followed by Liangshan Tibetan (ASLT, 0.0134) and Liangshan Yi (ASLY, 0.0146). The African AmaXhosa (SAAX) shows the largest genetic relationship with Nagqu Tibetan (0.2097).

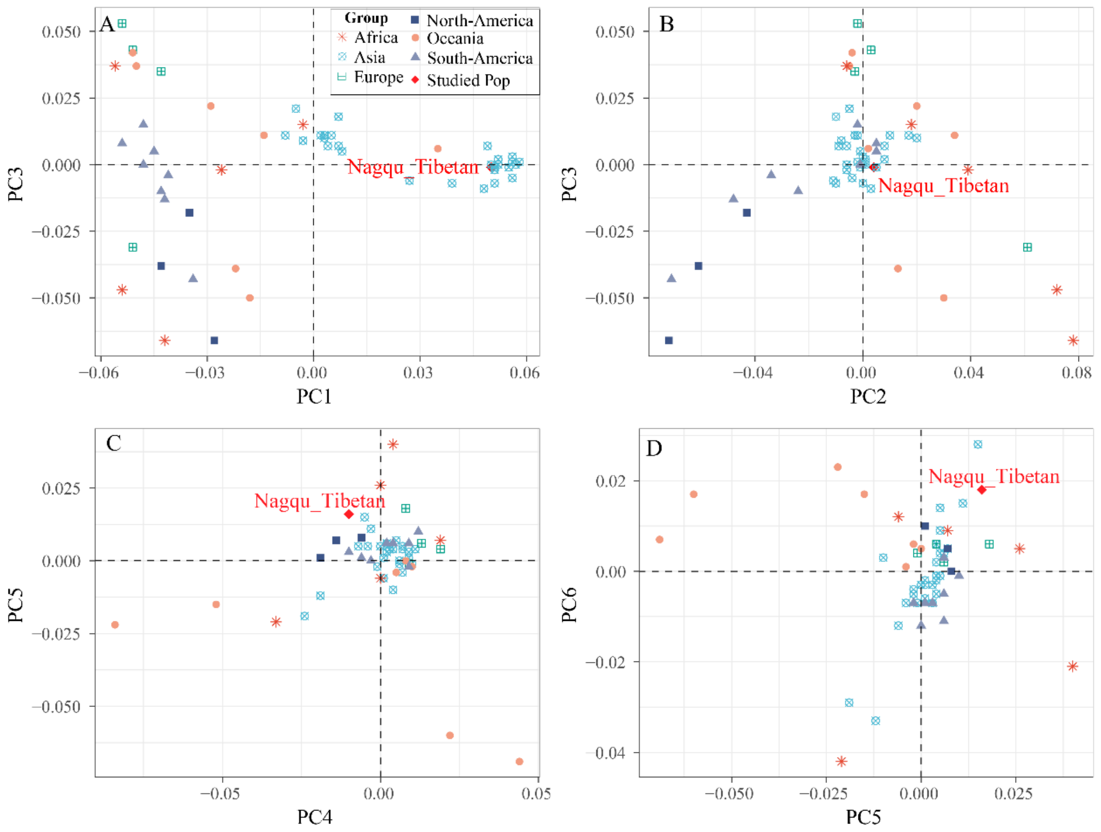

Figure S3. Population affinity between Nagqu Tibetan and other 56 worldwide reference populations inferred from the principal component analysis based on the allele frequency distributions.

The full population names are submitted in the Supplementary Table S1. PCA based on the top six components could explain 74.52% variance (PC1 to PC6: 34.18%, 14.49%, 11.70%, 6.16%, 5.00% and 2.99%).

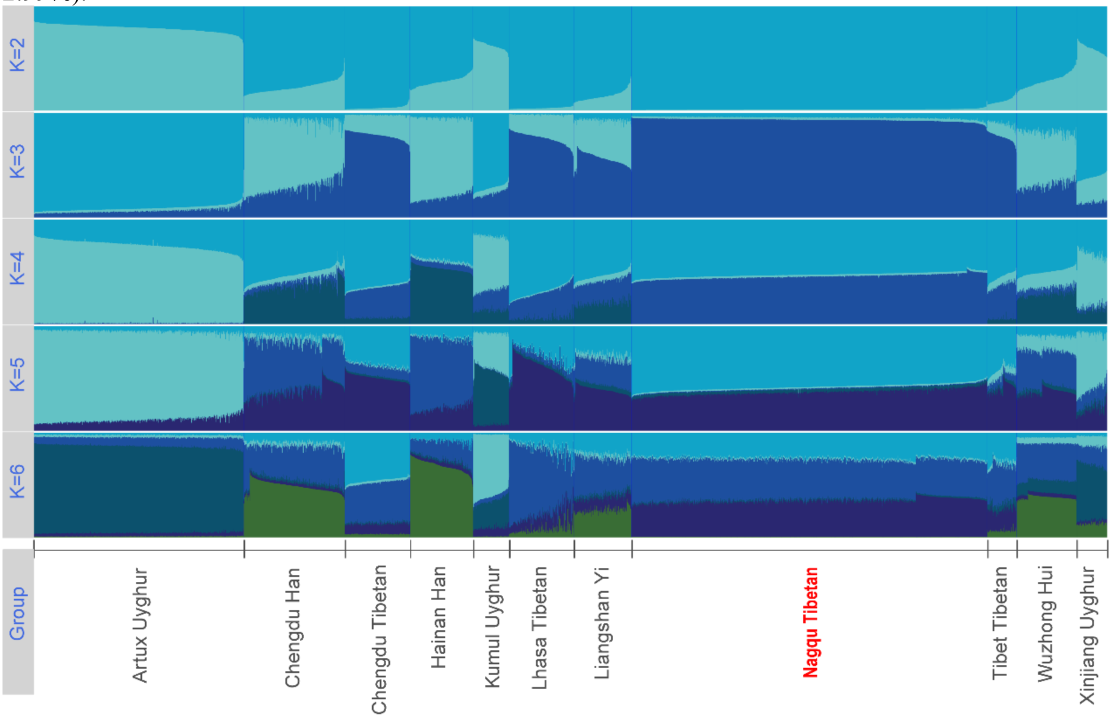

Figure S4. Shared composition of 3287 individuals from 11 Chinese reference populations inferred from the raw genotype data.

At K = 2, we identify two distinct components deriving from ST and AT populations. At K = 3, population substructures of Han Chinese and Tibeto-Burman (TB) populations are observed within ST populations. Geographically different components within the same language family gradually appear with the increase of K values and the proportions of shared components are variable within ethnically different groups. The optimal K value was K=3.

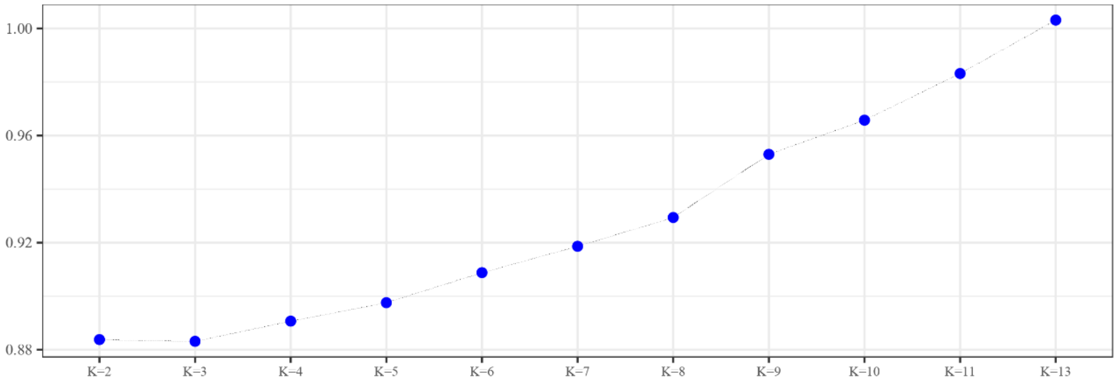

Figure S5. The distribution of cross-validation error value in model-based ADMIXTURE analysis with different predefined clusters.

K=3 with the least value of 0.8831.

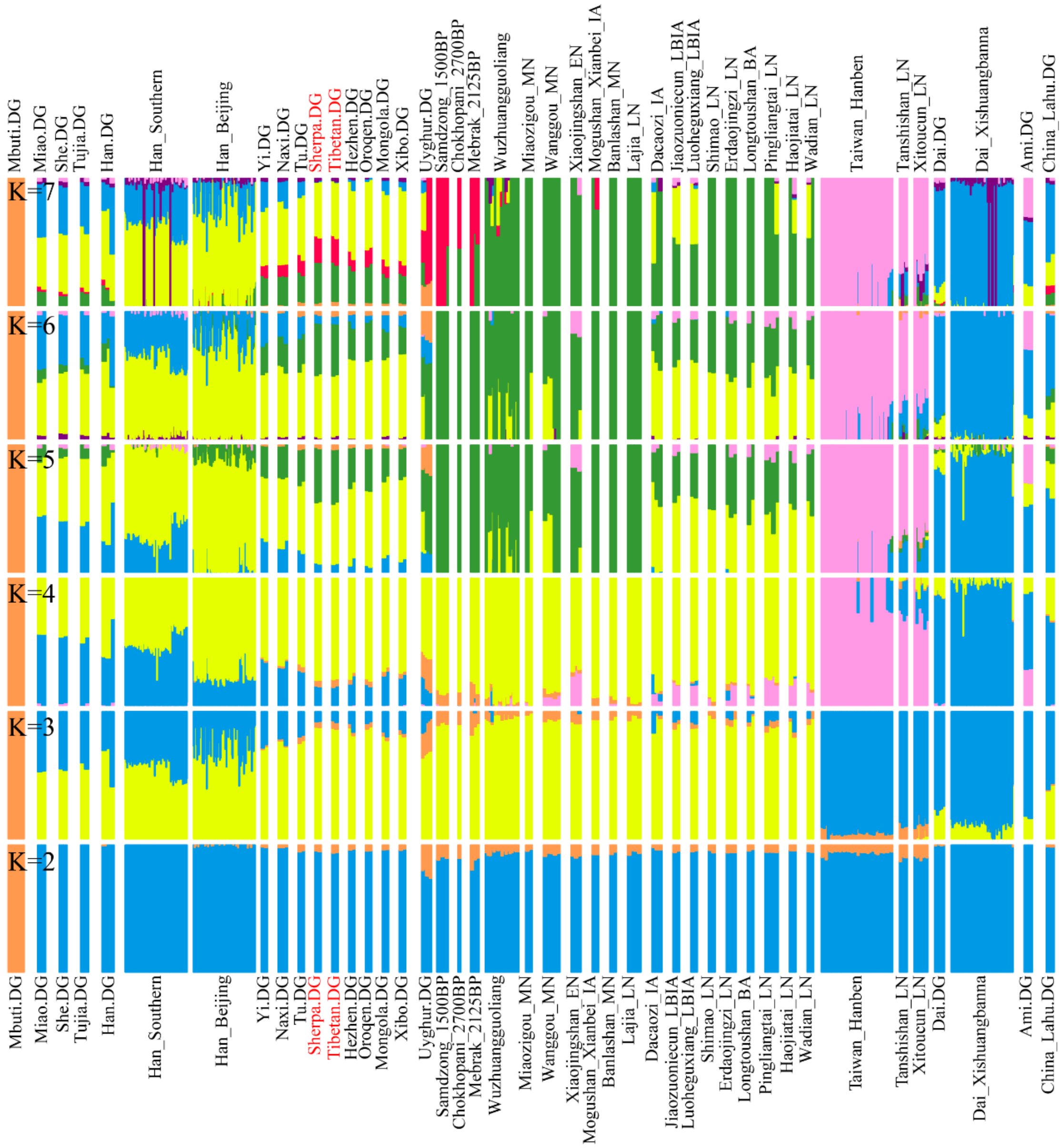

Figure S6. Individual-level shared composition in the model-based ADMIXTURE analysis. Results with 2 to seven predefined cluster sources were visualized here.

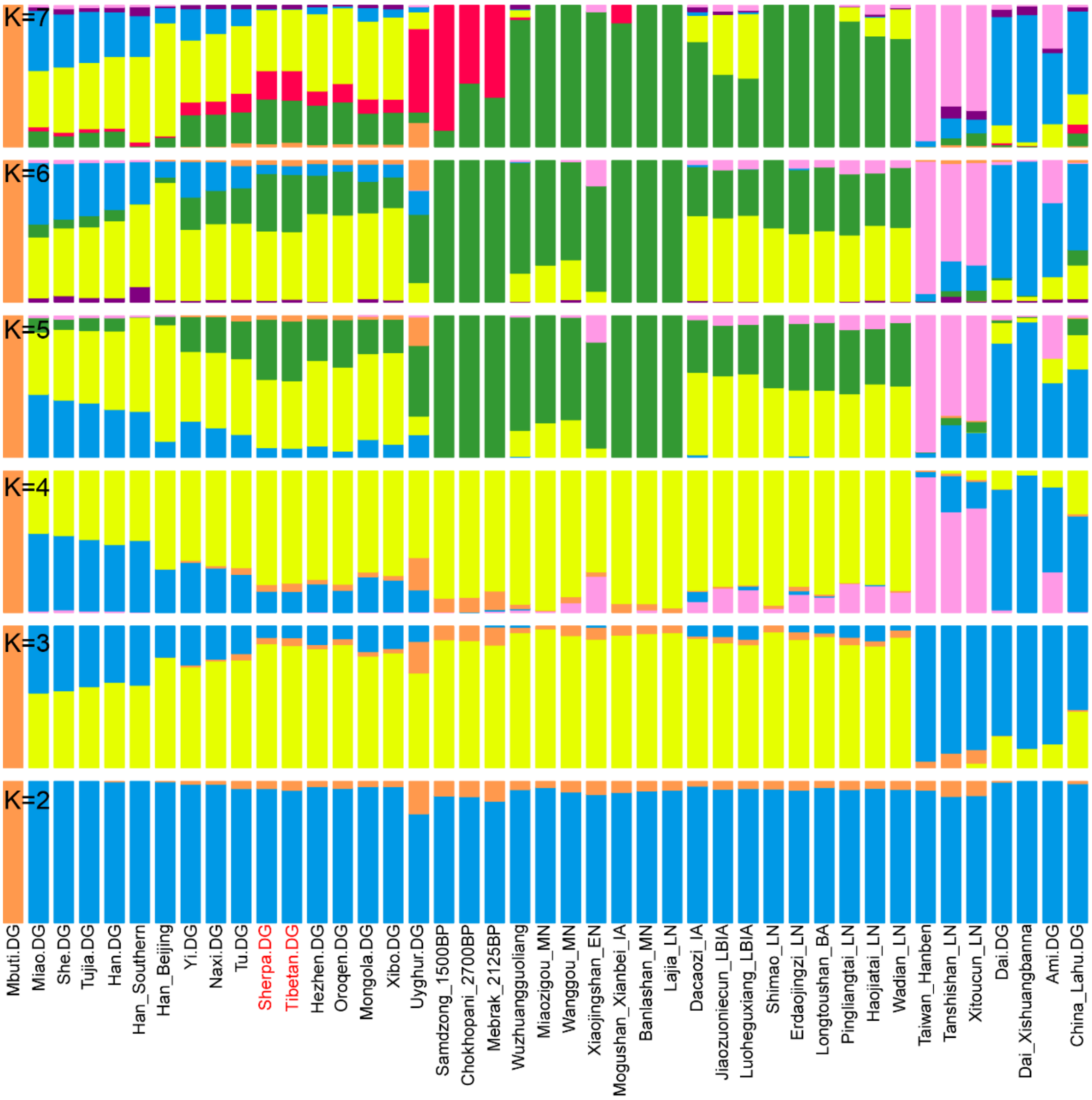

Figure S7. Population-level shared composition in the model-based ADMIXTURE analysis. Results with 2 to seven predefined cluster sources were visualized here.

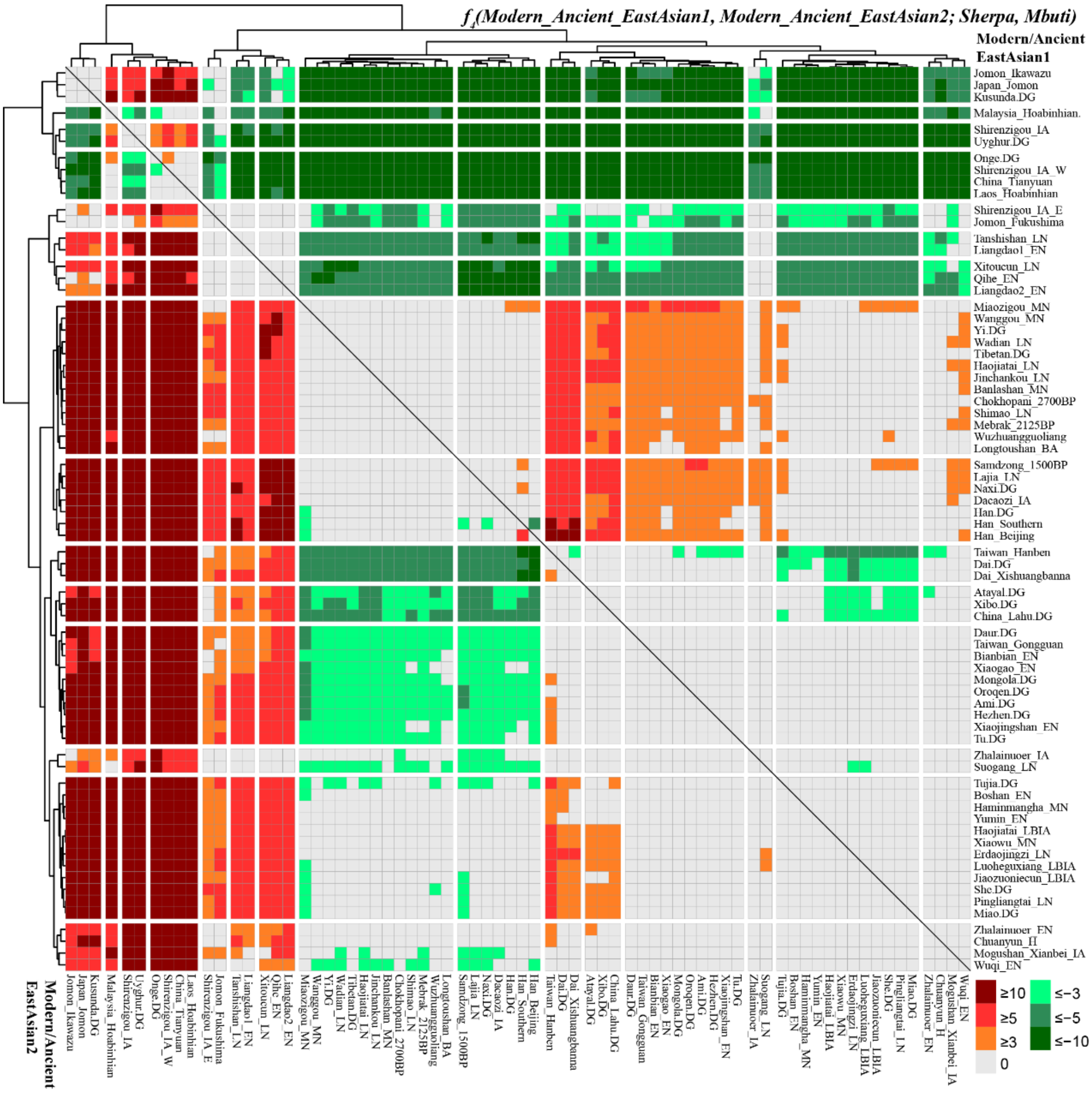

Figure S8. Shared genetic drift between highland East Asian Sherpa and other modern and ancient reference populations via f_4*(modern/ancient East Asian1, modern/ancient East Asian2; Sherpa, Mbuti)*.

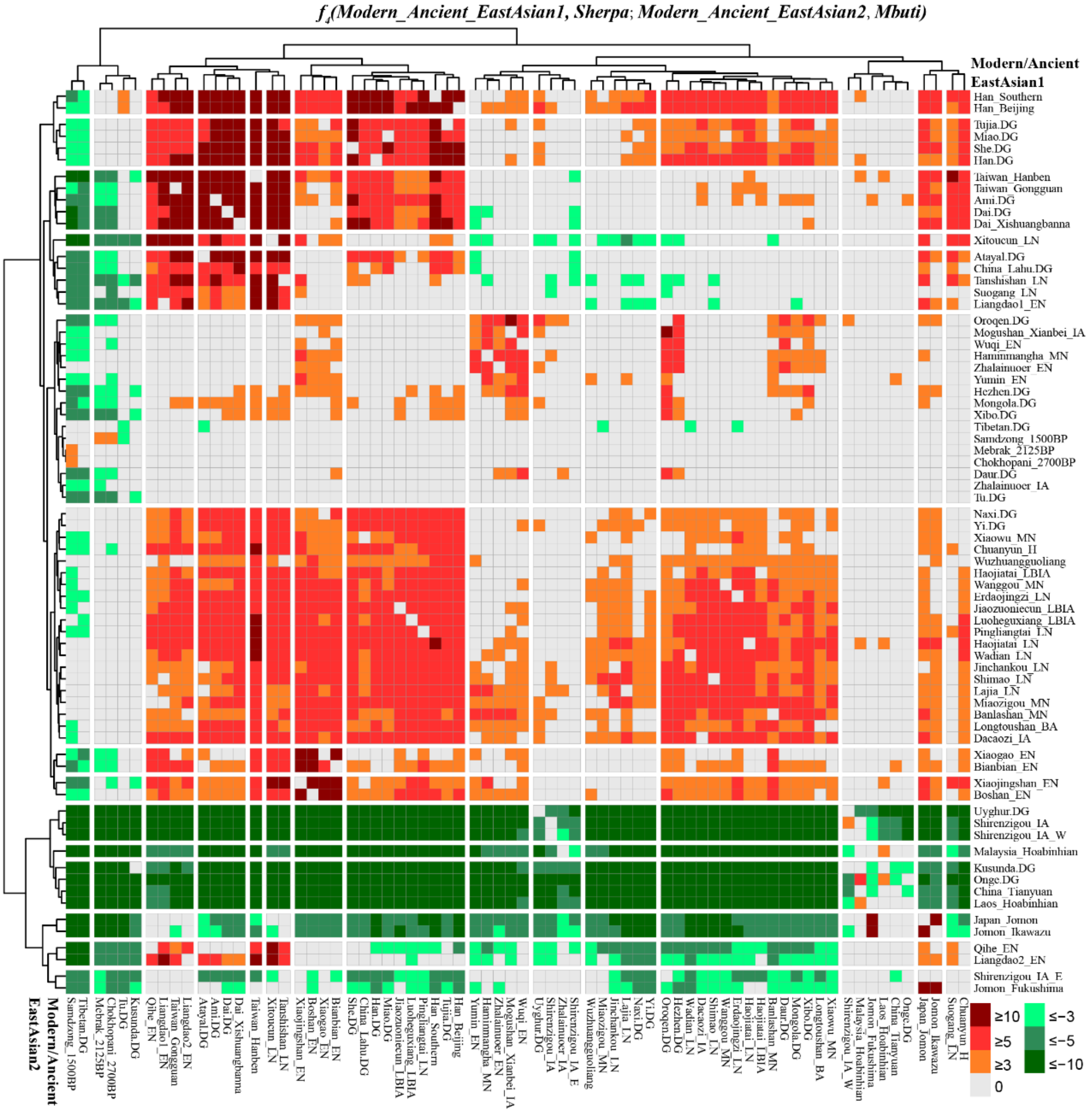

Figure S9. Shared genetic drift between highland East Asian Sherpa and other modern and ancient reference populations via *f*$_4$*(modern/ancient East Asian1, Sherpa; modern/ancient East Asian2, Mbuti)*.
